# Modelling the propagation of infectious disease via transportation networks

**DOI:** 10.1038/s41598-022-24866-3

**Published:** 2022-11-29

**Authors:** Anupriya, Prateek Bansal, Daniel J. Graham

**Affiliations:** 1 Transport Strategy Centre, Department of Civil and Environmental Engineering, Imperial College London, London, SW7 2AZ UK; 2 Department of Civil and Environmental Engineering, National University of Singapore, Queenstown, 119077 Singapore

**Keywords:** Infectious diseases, Statistics

## Abstract

The dynamics of human mobility have been known to play a critical role in the spread of infectious diseases like COVID-19. In this paper, we present a simple compact way to model the transmission of infectious disease through transportation networks using widely available aggregate mobility data in the form of a zone-level origin-destination (OD) travel flow matrix. A key feature of our model is that it not only captures the propagation of infection via direct connections between zones (first-order effects) as in most existing studies but also transmission effects that are due to subsequent interactions in the remainder of the system (higher-order effects). We demonstrate the importance of capturing higher-order effects in a simulation study. We then apply our model to study the first wave of COVID-19 infections in (i) Italy, and, (ii) the New York Tri-State area. We use daily data on mobility between Italian provinces (province-level OD data) and between Tri-State Area counties (county-level OD data), and daily reported caseloads at the same geographical levels. Our empirical results indicate substantial predictive power, particularly during the early stages of the outbreak. Our model forecasts at least 85% of the spatial variation in observed weekly COVID-19 cases. Most importantly, our model delivers crucial metrics to identify target areas for intervention.

## Introduction

Outbreaks of new or recurrent infectious diseases, particularly those with pandemic potential, pose unprecedented challenges to global public health and economy^1–3^. The ongoing COVID-19 pandemic, for instance, has not only claimed more than 6.5 million lives globally^4^, but also inflicted wide-ranging socio-economic costs (via unsettling labour supply and consumption), amounting to at least 12.5 trillion USD^5^. To contain the spread of such diseases, public health authorities seek models that can efficiently predict the spatio-temporal evolution of the disease in large geographical areas and inform effective pharmaceutical or non-pharmaceutical countermeasures (Pharmaceutical interventions include drug and vaccination delivery. Non-pharmaceutical measures comprise social distancing, quarantine measures and other infection control practices.). In most scenarios, with limited resources in hand, decision-makers desire a timely and precise identification of the target areas for intervention such that the overall socio-economic impact of the disease can be minimised^6–8^. The overarching aim of this study is to develop a simple compact model of infectious disease dynamics that can deliver metrics to inform such well-focused interventions.

Central to the development of such models is a description of human mobility, as increases in mobility are well-known to exacerbate the transmission of infectious diseases. Several previous studies have confirmed the positive association between mobility and propagation of pandemics and endemics in general, and COVID-19 in particular, offering insights at different spatial scales: continental, national, regional, and local. For instance, while some studies^7,9–11^ have explored the role of long-distance travel in the spread of infectious diseases, other studies^12–17^ have focused on dynamics of urban mobility and community transmission of infections. A previous study^18^ also investigated the interplay between short-distance commuting and long-distance travel in the importation of infections through multi-layered mobility networks. The propagation of infections has been analysed by exploiting various sources of mobility data, such as GPS data from mobile phones^13,19–22^, Google^17^, and social media platforms such as Facebook^23–25^ and Twitter^14^.

There are two prominent strands of the literature on analysis of infection transmission using mobility data. The first strand adopts a detailed epidemiological model, such as the susceptible-exposed-infectious-removed (SEIR) model, to simulate the importation of infections through mobility networks^26–28^. Such classic models, however, require various assumptions on population mixing, population compartment sizes and viral attributes such as incubation period^21^. The second strand of the literature leverages data on mobility patterns, mostly in real-time, to construct short-term forecasts of infection transmission risk^19–21,24,29^. In particular, such studies focus on the correlation between population flows, often represented in the form of aggregated origin-destination (OD) travel interactions between different locations in a given period, and the spatial distribution of infections in the subsequent period.

In this paper, we contribute to the second strand of the literature by reassessing and improving the utility of aggregated mobility data to model the potential propagation of infectious diseases through transportation networks. Specifically, we argue that a limitation of previous analytical studies^19,21,24^ is that they have restricted their analyses to the role of *direct* flows between the infected locations, say location *i*, and any other locations, say location *j*, in determining the diffusion of infections. In addition to these direct or first-order effects, we emphasise that the propagation of infectious diseases can also occur through subsequent interactions in the transport system or higher-order effects. For instance, transmission first occurs because infection at location *i* is delivered to locations *j* via direct connections, but subsequently, infection propagates to locations *k* through their interactions with *j*, and further to locations *l* through their interactions with *k*, and so on. Accordingly, we reformulate the infection propagation process and propose an *inverse connectivity matrix* (ICM) to model this potential transmission of infections that captures both first- and higher-order connectivity effects. The resulting ICM is derived from an inverted origin-destination (OD) matrix. We further estimate a location-specific *connectivity propagation metric* (CPM) from the ICM, the value of which signifies the total potential transmission of the infection in the system as a consequence of an additional infection in that location. This metric can help public health authorities identify target areas for intervention, particularly during the early stages of disease outbreaks or resurgences, to alleviate the total impact of the disease on the system.

We note that the closest antecedent to our analysis is a previous study^30^ that demonstrates via a simulation exercise that the diffusion of infections through an air-transportation network is not limited to the direct path (or the most probable path) between two nodes, but other indirect paths in the network also play a significant role in the diffusion process. Another related study^31^ also considered the idea of multiple possible paths to locate the source of large-scale outbreaks of food-borne disease. The notion of multiple possible paths has also been considered in modelling the spread of information between two nodes in communication networks^32^. However, we find that most analytical studies continue to model the disease propagation process via the most probable path only, apparently due to the simplicity of deriving these direct effects from observed OD flows. In this study, we demonstrate that a simple inversion of the OD matrix results in a matrix that captures the indirect paths in addition to the direct paths of transmission. The elements of the proposed inverted OD matrix can replace direct OD flows to model the propagation of infectious diseases via transportation networks.

To show how our proposed matrix (that is, ICM) models the potential spread of infectious diseases through transportation networks, we simulate a synthetic OD matrix. The matrix is designed to best approximate the observed distribution of real-world long-distance trips. The simulation demonstrates the importance of capturing higher-order connectivity effects in modelling propagation. We then evaluate the performance of our proposed matrix empirically using zone-level OD data and COVID-19 caseload data from (i) Italy and (ii) the New York Tri-State area. The OD data have been estimated in previous studies^22,33^ from large-scale real-time mobility data of geo-located smartphone users. We use the mobility data to examine the correlation of our model forecasts with the observed zone-level (province-level in Italy and county-level in New York Tri-State area) case data in future time periods. Our empirical results indicate substantial predictive power with more than 85 percent of the spatial variation in observed weekly zonal COVID-19 cases being predicted by our model. We emphasise that similar to previous studies in this strand of the literature, we focus solely on predicting the transmission risk based on mobility patterns without precisely predicting future cases, which would require a detailed epidemiological model such as the SEIR model.

The remainder of this paper is organised as follows. The next section sets out the proposed analytical framework to model mobility and infections, which is followed by the simulation study. The penultimate section presents empirical case studies of COVID-19 propagation in Italy and the Tri-State area. Conclusions and recommendations are drawn in the final section.

## An inverse origin-destination (OD) matrix to model infection propagation

Previous studies^34^ have shown that in a model of infectious disease dissemination through transportation networks, epidemiological factors enter the infection dynamics independently from the mobility parameters. Accordingly, we first develop a mobility model that captures first and higher-order connectivity effects. Thereafter, we supplement the model with epidemiological inputs to translate the connectivity effects into infections.

We consider a typical OD matrix with the following layout.

$$\begin{aligned} \bordermatrix {&1&2&3&4&\cdots&n \\1&x_{11}&x_{12}&x_{13}&x_{14}&\ldots&x_{1n} \\2&x_{21}&x_{22}&x_{23}&x_{24}&\ldots&x_{2n} \\3&x_{31}&x_{32}&x_{33}&x_{34}&\ldots&x_{3n} \\4&x_{41}&x_{42}&x_{43}&x_{44}&\ldots&x_{4n} \\\vdots&\vdots&\vdots&\vdots&\vdots&\ddots&\vdots \\n&x_{n1}&x_{n2}&x_{n3}&x_{n4}&\ldots&x_{nn} \\} \end{aligned}$$

The cells of the OD matrix contain values ($x_{ij}$) that measure transportation interactions between zones *i* and *j* in units of traffic volume such as trips. Reading across the rows of the matrix indicates trips that originate in zone *i*, $i=(1,\ldots ,n)$, and end in *j*, $(j=(1,\ldots ,n)$, while the columns show trips ending in *j* from each zone *i*. For any origin *i*, the sum of elements along the corresponding row of the matrix gives total trips originating in *i*, $O_i=\sum _{j=1}^n x_{ij}$, while for any destination *j*, the corresponding column-sum gives total trips ending in *j*, ${D_j}=\sum _{i=1}^n x_{ij}$.

We are interested in using the matrix to model the potential propagation of infectious diseases like COVID-19 via transport networks. Given the presence of infection in the system, our assumption is that the potential infectious interactions (henceforth, interactions) generated in a zone, say zone *i*, will be determined by:

1. **Zonal activity scale**—the scale of general interactions that occur between residents and non-residents active within the zone. For any zone *i*, the zonal activity scale $t_i$ can be proxied using the total trips originating and ending in that zone as $t_i = (O_i+D_i)/2$. In most empirical applications, it can be shown that $t_i \approx O_i$.
2. **Zonal connectivity**—the interaction that occurs via transport network connectivity. Crucially, for this component, we are concerned with both *first-order* and *higher-order* connectivity effects.

Following from the above assumptions, we propose an interaction generation-propagation model shown in Table 1. This table illustrates the dynamics of diffusion of interactions in the system. One may note from this table that the total interactions generated in any zone *i* (that is, the sum of trips ended and zonal activity scale) equals the total interactions propagated by that zone (measured by the sum of trips originated and trips imported). As we describe in the rest of this section, this table fulfils two separate functions. First, it is a descriptive framework for showing the relationship between the interactions originating and ending in each zone and between inputs given by the zonal activity scale and outputs given by the interactions propagated in the system. Second, it provides us with an analytical tool for measuring the impact of interventions on the total interactions propagated in the system.

**Table 1:** Interaction transmission table.

From Origins	To Destinations						Interaction originated	Interaction imported	Zonal propagation potential
	1	2	3	4	$\ldots$	*n*			
1	$x_{11}$	$x_{12}$	$x_{13}$	$x_{14}$	$\ldots$	$x_{1n}$	$\sum _{j=1}^n x_{1j}$	${D_1}$	$p_1$
2	$x_{21}$	$x_{22}$	$x_{23}$	$x_{24}$	$\ldots$	$x_{2n}$	$\sum _{j=1}^n x_{2j}$	${D_2}$	$p_2$
3	$x_{31}$	$x_{32}$	$x_{33}$	$x_{34}$	$\ldots$	$x_{3n}$	$\sum _{j=1}^n x_{3j}$	${D_3}$	$p_3$
4	$x_{41}$	$x_{42}$	$x_{43}$	$x_{44}$	$\ldots$	$x_{4n}$	$\sum _{j=1}^n x_{4j}$	${D_4}$	$p_4$
$\vdots$	$\vdots$	$\vdots$	$\vdots$	$\vdots$	$\ddots$	$\vdots$	$\vdots$	$\vdots$	$\vdots$
n	$x_{n1}$	$x_{n2}$	$x_{n3}$	$x_{n4}$	$\ldots$	$x_{nn}$	$\sum _{j=1}^n x_{nj}$	${D_n}$	$p_n$
Total interaction ended	$\sum _{j=1}^n x_{j1}$	$\sum _{j=1}^n x_{j2}$	$\sum _{j=1}^n x_{j3}$	$\sum _{j=1}^n x_{j4}$	$\ldots$	$\sum _{j=1}^n x_{jn}$	-	$D=\sum _{i=1}^n {D_i}$	-
Zonal activity scale	${\frac{O_1+D_1}{2} \approx O_1}$	${\frac{O_2+D_2}{2} \approx O_2}$	${\frac{O_3+D_3}{2} \approx O_3}$	${\frac{O_4+D_4}{2} \approx O_4}$	$\ldots$	${\frac{O_n+D_n}{2} \approx O_n}$	$O=\sum _{i=1}^n {O_i}$	-	-
Zonal generation potential	$p_1$	$p_2$	$p_3$	$p_4$	$\ldots$	$p_n$	-	-	$p=\sum _{i=1}^n p_i$

From Table 1, we write the total propagation potential of any zone *i* as

1 $$\begin{aligned} p_i=t_i+\sum _{j=1}^n x_{ji}. \end{aligned}$$

The first term on the right-hand side of this equation represents the zonal activity scale. The second term, the sum of the elements of $i{th}$ column in the OD matrix, captures potential propagation via first-order connectivity to zone *i* from all other zones *j*.

Notice that the transmission dynamics formulated in Eq. (1) do not appear to capture higher-order effects. To achieve this we re-formulate the model. We define $a_{ij}=x_{ji}/p_j$ as a set of connectivity coefficients:

$$\begin{aligned} \bordermatrix {&1&2&3&4&\cdots&n \\1&a_{11}&a_{12}&a_{13}&a_{14}&\ldots&a_{1n} \\2&a_{21}&a_{22}&a_{23}&a_{24}&\ldots&a_{2n} \\3&a_{31}&a_{32}&a_{33}&a_{34}&\ldots&a_{3n} \\4&a_{41}&a_{42}&a_{43}&a_{44}&\ldots&a_{4n} \\\vdots&\vdots&\vdots&\vdots&\vdots&\ddots&\vdots \\n&a_{n1}&a_{n2}&a_{n3}&a_{n4}&\ldots&a_{nn} \\}. \end{aligned}$$

Note that the above matrix is essentially a transpose of the OD matrix, with every element $x_{ji}$ normalised by their corresponding $p_j$. The cells of this matrix, $a_{ij}$, measure the interactions transmitted from zone *j* to *i* as a proportion of the total interactions propagated by zone *j*. We emphasise that previous analytical studies have used analogous measures of connectivity such as the proportion of population flux^35^ from *j* to *i* and *effective distance*^34,36^ (effective distance, $d{ij} \approx 1 - \log a_{ji}$), to model the evolution of various diseases in networks via the most probable (direct) paths.

We can then write Eq. (1) above as:

$$\begin{aligned} p_i=\sum _{j=1}^n a_{ij} p_j+t_i. \end{aligned}$$

Using matrix notation to represent the whole system, we have:

$$\begin{aligned} \mathbf{{p}}=\mathbf{{A}}\mathbf{{p}}+\mathbf{{t}}, \end{aligned}$$

where $\mathbf{{p}}$ and $\mathbf{{t}}$ are column vectors of total zonal propagation potential and zonal scale respectively, and $\mathbf{{A}}$ is a $n \times n$ matrix of connectivity coefficients. Using the identity matrix $\mathbf{{I}}$, we can write

$$\begin{aligned} (\mathbf{{I}}-\mathbf{{A}})\mathbf{{p}}=\mathbf{{t}}, \end{aligned}$$

allowing us to derive total potential system propagation via the following expression:

2 $$\begin{aligned} \mathbf{{p}}=\left( \mathbf{{I}}- \mathbf{{A}}\right) ^{-1} \mathbf{{t}}. \end{aligned}$$

Expanding the right-hand side of Eq. (2) we can show how we capture higher-order effects in this re-formulation:

3 $$\begin{aligned} \left( \mathbf{{I}}- \mathbf{{A}}\right) ^{-1}\mathbf{{t}}= \mathbf{{t}}+\mathbf{{A}}\mathbf{{t}}+\mathbf{{A}}^2\mathbf{{t}}+\mathbf{{A}}^3\mathbf{{t}}+\mathbf{{A}}^4\mathbf{{t}}+\cdots \end{aligned}$$

The first term in this expansion captures interactions generated via zonal scale, the second term captures first-order propagation to any zone via transport network connectivity (or equivalently, via the most probable paths in the network), the third term captures second-order propagation generated via the first-order effects, term four is third-order effects generated by the second-order connectivity, and so on. In particular, we note that the cells of the matrix $\left( \mathbf{{I}}- \mathbf{{A}}\right) ^{-1} - \mathbf{{I}}\approx \mathbf{{A}}^{-1}$, say $b_{ij}$, measure successive interaction effects from first and higher order connectivity between zone *i* and zone *j*, as shown below:

$$\begin{aligned} \begin{aligned} b_{ij}&= a_{ij} + \sum _{k=1}^n a_{ik} a_{kj} + \sum _{k=1}^n \sum _{l=1}^n a_{il} a_{lk} a_{kj} + \hdots = \frac{x_{ji}}{p_j} + \sum _{k=1}^n \frac{x_{jk}}{p_j} \frac{x_{ki}}{p_k} + \sum _{k=1}^n \sum _{l=1}^n \frac{x_{jk}}{p_j} \frac{x_{kl}}{p_k} \frac{x_{li}}{p_l} + \hdots \\ \end{aligned} \end{aligned}$$

The inverse matrix

$$\begin{aligned} \mathbf{{A}}^{-1} = \mathbf{{A}}+ \mathbf{{A}}^2 + \mathbf{{A}}^3 + \mathbf{{A}}^4 + \hdots \end{aligned}$$

is thus of key interest for our calculations. We refer to this matrix as the *inverse connectivity matrix* (ICM).

We now supplement our model with epidemiological inputs to calculate metrics that indicate the relative prevalence of infections in each zone of the system. To do so, we first construct a $n \times 1$ vector $\mathbf{{R}}$, with elements $R_j$ representing the relative strength of the infectious disease in the corresponding zone *j*. We adopt a heuristic approach to measure $R_j$ based on qualitative information about epidemiological factors via active case counts, compounded by a measure of total activity in the zone. We measure $R_j$ as the proportion of active cases in zone *j* relative to its population, times the zonal activity scale $t_j$, that is,

$$\begin{aligned} R_j= \left( \frac{\hbox { Active cases in zone}\ j}{\displaystyle \sum _{j=1}^n \hbox { Active cases in zone}\ j} \Bigg / \frac{{\text {Population of zone }}\, j}{\displaystyle \sum _{j=1}^n \text {Population of zone }\, j} \right) \times t_j. \end{aligned}$$

We also define a $n \times 1$ vector $\mathbf{{S}}$ of zonal susceptibility capturing the number of susceptible individuals in each zone. We calculate zonal susceptibility, $S_i$, of the corresponding zone *i* as the population of zone *i* minus the number of individuals immune to the disease in zone *i*, that is,

$$\begin{aligned} S_i=\text {Population of zone } i-\hbox { Number of immune individuals in zone}\ i. \end{aligned}$$

Accordingly, we construct the *relative infection prevalence metric* (IPM) as follows:

4 $$\begin{aligned} \text {IPM} = (\text {ICM} \times \mathbf{{R}}) \odot \mathbf{{S}}\end{aligned}$$

where $\odot$ represents the Hadamard or element-wise product of the associated vectors. The metric $\text {IPM}_i$ for each zone *i* characterises the aggregate strength of mixing between infected travellers from all zones $j, j = 1, 2, \ldots , n$ and susceptible hosts in *i*. We represent the metric $\text {IPM}_i$ in unit notation to illustrate its significance:

$$\begin{aligned} \begin{aligned} \text {IPM}_i = S_i\sum _{j=1}^n b_{ij}R_j&= S_i \sum _{j=1}^n R_j \left( a_{ij} + \sum _{k=1}^n a_{ik} a_{kj} + \sum _{k=1}^n \sum _{l=1}^n a_{il} a_{lk} a_{kj} + \hdots \right) \\&= S_i \sum _{j=1}^n R_j \left( \frac{x_{ji}}{p_j} + \sum _{k=1}^n \frac{x_{jk}}{p_j} \frac{x_{ki}}{p_k} + \sum _{k=1}^n \sum _{l=1}^n \frac{x_{jk}}{p_j} \frac{x_{kl}}{p_k} \frac{x_{li}}{p_l} + \hdots \right) .\\ \end{aligned} \end{aligned}$$

Note that the $i{th}$ term of the column vector IPM represents the summation of first-order and higher-order infection transmission effects from all zones *j* to *i*, multiplied by the zonal susceptibility $S_i$. Intuitively, this term sums the contribution of infections in each zone $j, j=1,2,\ldots n$ towards infections in zone *i* generated via direct and indirect importation of infections from zone *j* to zone *i*. In other words, the metric sums up the infection effects propagated to zone *i* via all possible paths in the system. This metric can be matched with the observed raw case counts in the zone to assess the performance of the model.

Furthermore, for each zone *i*, note that the sum $\sum _j b_{ji}S_j$, amplified by the corresponding zonal activity scale $t_i$, measures the sum of infectious effects propagated in the system as a result of an additional infection in zone *i*. We refer to the resulting metric as the *connectivity propagation metric* (CPM), which is given by:

5 $$\begin{aligned} \begin{aligned} \text {CPM}_i=t_i\sum _{j=1}^n b_{ji}S_j&= t_i \sum _{j=1}^n S_j \left( a_{ji} + \sum _{k=1}^n a_{jk} a_{ki} + \sum _{k=1}^n \sum _{l=1}^n a_{jk} a_{kl} a_{li} + \hdots \right) \\&= t_i \sum _{j=1}^n S_j \left( \frac{x_{ij}}{p_i} + \sum _{k=1}^n \frac{x_{ik}}{p_i} \frac{x_{kj}}{p_k} + \sum _{k=1}^n \sum _{l=1}^n \frac{x_{il}}{p_i} \frac{x_{lk}}{p_l} \frac{x_{kj}}{p_k} + \hdots \right) .\\ \end{aligned} \end{aligned}$$

The higher the CPM for any zone, the higher the potential number of infections in the system due to an infection in the zone and thus the higher the need for an intervention in that zone. The CPM of zone *i* can be multiplied by the number of active cases in the zone to estimate the net impact of all infections in zone *i* on the system.

In the Appendix, we present a summarised algorithm to implement the proposed model.

## Simulation

**Figure 1:**
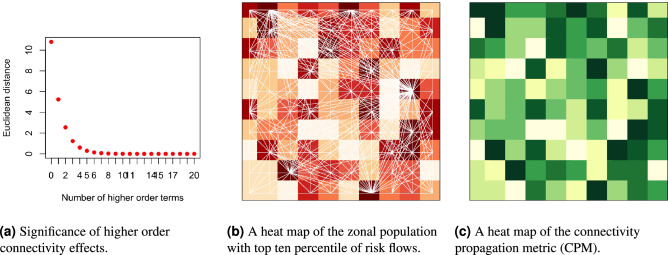
A simulation study to demonstrate the potential propagation of interactions via transportation network connectivity.

In this section, we present a brief simulation study to evaluate how the ICM models the potential propagation of infectious interactions via transport networks, and specifically, to investigate the potential significance of higher-order effects in the propagation process. We consider a transportation network comprising one hundred zones, each indexed by *i*, $i=(1,\ldots ,100)$, with a layout as shown in Fig. 1b. The population of each zone is drawn from a log-normal distribution with mean 1000 and standard deviation 800. To simulate the corresponding OD matrix, we adopt the radiation model for human mobility^37^. This model is widely known to best approximate the observed distribution of real-world long-distance trips. The fundamental equation of the radiation model quantifies the average flux or trips, $X_{ij}$, from zone *i* to zone *j*:

6 $$\begin{aligned} X_{ij} = X_i \times \frac{m_i \times n_j}{(m_i+q_{ij})(m_i+n_j+q_{ij})} \end{aligned}$$

where $X_i$ is the total number of travellers from zone *i*, $m_i$ and $n_j$ are the population in zone *i* and *j* respectively, and $q_{ij}$ is the total population in the circle centered at *i* and touching *j* excluding the source and the destination population. We generate the values of $X_i$, that is, the total number of travellers from zone *i*, by assuming the proportion of travellers in each zone to be uniformly distributed in a range [0.5, 1]. We set the susceptibility $S_i$ of each zone *i* as 1. After generating the OD matrix with terms $X_{ij}$, we estimate other quantities ($\mathbf{{t}}$ vector, $\mathbf{{p}}$ vector, $\mathbf{{A}}$ matrix, ICM, and CPM vector) as explained in the previous section.

To understand the contribution of higher-order transport connectivity effects towards the zonal propagation potential $\mathbf{{p}}$, we obtain approximate estimates of $\mathbf{{p}}$ (that is, $\left( \mathbf{{I}}- \mathbf{{A}}\right) ^{-1} \mathbf{{t}}$) by iteratively adding one higher-order term at a time. At each iteration *l*, we estimate $\mathbf{{p}}$ using Eq. (7).

7 $$\begin{aligned} \mathbf{{p}}= \left( \mathbf{{I}}- \mathbf{{A}}\right) ^{-1} \mathbf{{t}}= \sum _{r=0}^{l} \mathbf{{A}}^r\mathbf{{t}}\end{aligned}$$

We record the euclidean distance between the observed $\mathbf{{p}}$ and the approximated $\mathbf{{p}}$ with varying higher-order effects at each iteration. Figure 1a plots these euclidean distance values over the number of higher-order terms included in the approximation. This figure illustrates that higher-order effects up to the fifth degree contribute significantly to the observed total propagation potential of the system. Note that the importance of higher-order effects inherently depends on the data-generating process of the empirical study and can vary across applications and geographical settings.

Figure 1b shows a heat map of the zonal population with the top ten percentile of flows $X_{ij}$. Figure 1c shows the corresponding heat map of the CPM. We observe that zones with higher intensity of flows are by and large associated with a higher CPM value. Note that figure 1b shows only the first-order propagation via transport network connectivity and does not include any higher-order effects, so there may not be a one-to-one correspondence between the figures. Based on this figure, we conclude that the higher the CPM value for a zone, the higher the number and intensity (that is, the proportion of trips) of connections between the zone and the entire system, and as a result, the higher will be the impact of interactions propagating from the zone on the entire system. As discussed in the previous section, we can complement these interactions with active caseload data to model the potential propagation of infectious diseases like COVID-19 through transport networks.

## Case Studies

To gauge the predictive performance of the proposed matrix, we consider the first wave of COVID-19 infections in (i) Italy and (ii) the New York Tri-State (New York, New Jersey and Connecticut) area. The earliest cluster of cases in Italy were detected in Lombardy and Veneto on February 21, 2020 and by the beginning of March 2020, the SARS-CoV-2 virus had spread to all Italian provinces. On March 9, 2020, a nation-wide lockdown was announced in Italy, which was gradually eased in May 2020. By June 3, 2020, freedom of movement across Italian regions and other European countries was restored. The first case in the Tri-State area was confirmed on March 1, 2020 and the region subsequently emerged as an epicenter of the pandemic in the United States. A full lockdown was imposed in the region between March 20, 2020 to May 15, 2020, which was followed by a phased reopening of regions (contingent upon case counts) through the latter half of May and June 2020.

Based on the above-discussed series of events, we consider the period between March 2020 to May 2020 as the study period for Italy. For this period, we investigate correlations between the observed weekly raw COVID-19 case counts in each Italian province and the IPM of each province estimated using observed mobility patterns and active case counts in the preceding week. A lag of one week between mobility patterns and raw case counts allows us to account for the transition time between exposure to case detection. Similarly, the period between March 2020 to June 2020 is the study period for the Tri-State area, where we explore the correlations between weekly case counts and estimated IPM at the county level.

### Data

We use a publicly available mobility dataset comprising daily origin-destination (OD) movements of the population between Italian provinces for the period January 18, 2020, to June 26, 2020. These OD matrices have been computed by Pepe et al. (2020)^22^ using large-scale GPS data of about 170,000 de-identified smartphone users provided by Cuebiq Inc, a location intelligence and measurement platform. We obtain similar OD matrices for the mobility in the Tri-State area from another public database^33^. The data consists of a record of daily OD travel flows between counties in the United States since January 1, 2019, constructed by analysing the trajectories of millions of mobile phone users as provided by SafeGraph, a global geospatial data company.

Additionally, we obtain COVID-19 epidemiological data for Italy from a public repository maintained by Il Dipartimento della Protezione Civile, which has also been used in previous studies^38^. Data on daily COVID-19 cases in the Tri-State Area counties is obtained from a similar platform that records data published by state public health agencies in the US. The two caseload datasets comprise a diurnal record of the cumulative numbers of positive COVID-19 cases in each zone (Italian province or Tri-State area county). From these cumulative numbers, we calculate the daily number of new cases in each zone. Note, however, that the data do not provide the daily numbers of active cases. We, therefore, use the daily cumulative number of cases in each zone *i* as a proxy for the daily active case counts in that zone to estimate the relative strength of infection $R_i$. This approximation presumes the daily active caseload in each zone to be a certain time-varying percentage, $k_t$, of the cumulative number of cases observed each day *t*, which does not vary substantially across zones. Thus, $R_j$ can be approximated as:

$$\begin{aligned} R_j\approx & {} \left( \frac{k_t \times \hbox { Cumulative cases in zone}\ j}{\displaystyle \sum _{j=1}^n k_t \times \hbox { Cumulative cases in zone}\ j} \Bigg / \frac{\hbox { Population of zone}\ j}{\displaystyle \sum _{j=1}^n \hbox { Population of zone}\ j} \right) \\\times & {} t_j = \left( \frac{\text {Cumulative cases in zone }\, j}{\displaystyle \sum _{j=1}^n \text {Cumulative cases in zone }\, j} \Bigg / \frac{\text {Population of zone }\, j}{\displaystyle \sum _{j=1}^n \hbox { Population of zone}\ j} \right) \times t_j \end{aligned}$$.

Further, we estimate zonal susceptibility $S_i$ by subtracting the cumulative case count of zone *i* from its estimated population. Note that existing evidence^39^ suggests that COVID-19 infection-induced immunity lasts for at least six months. Accordingly, our $S_i$ estimate assumes that individuals once infected by the disease during our study period (< 6 months long) develop immunity against subsequent reinfections in this period. Data on population size for each Italian province is obtained from the official estimates provided by Il Istituto Nazionale di Statistica, ISTAT. County-level population estimates for the Tri-State area are extracted from the US Census Bureau website.

## Results

This section has four sub-sections. The first subsection presents a brief precursor to our main analysis where we assess the independent role of mobility data (that is, without epidemiological inputs) in determining the future spatial distribution of COVID-19 infections. The second subsection assesses the performance of the proposed model by investigating the correlations between the prevalence metric (IPM) and the raw case counts in the subsequent period. The penultimate subsection investigates the role of higher-order connectivity effects in disease propagation. In the final section, we use our model estimates are used to identify the top zones for any likely intervention.

### Preliminary analysis

In general, the raw case incidences in any zone *i* in week *t*, say $I_{i,t}$, is a first-order auto-regressive process of the infections in that zone. We input a network connectivity indicator (NCI) in an auto-regressive model of infections in zone *i* (Eq. (8)) to understand its significance in the infection generation process. The purpose of the NCI is to capture interaction effects propagated to *i* in week $t-1$ via all possible paths in the network. We construct NCI as $\text {NCI}=\text {ICM} \times \mathbf{{t}}$ using mobility data for week $t-1$. Essentially, $\text {NCI}_{i,t-1} = \sum _{j=1}^n b_{ij}t_j$ sums the effect of transportation interactions between zones *i* and all zones $j; j=1,\ldots ,n$ in week $t-1$ towards new infections generated in *i*, assuming all interactions to carry an identical risk of infection. We expect its effect on the dependent variable in Eq. (8) to be positive.

8 $$\begin{aligned} I_{i,t}= \alpha I_{i,t-1} + \beta \text {NCI}_{i,t-1} + \varepsilon _{i,t} \end{aligned}$$

where, $\alpha$ and $\beta$ are the model parameters. $\varepsilon _{i,t}$ is an idiosyncratic error term representing all random shocks to the dependent variable. The results from ordinary least squares estimation of Eq. (8) are presented in Table 2.

**Table 2:** Summary of results from the auto-regression infection generation model.

Explanatory variable	Italy		Tri-State area	
	Coefficient	Std.Error	Coefficient	Std.Error
Lag of raw case incidence (dependent variable)	$8.55 \times 10^{-1}$***	$1.40 \times 10^{-2}$	$8.69 \times 10^{-1}$***	$1.48 \times 10^{-2}$
Network connectivity indicator (NCI)	$3.39 \times 10^{-5}$***	$6.24 \times 10^{-6}$	$9.73 \times 10^{-5}$***	$2.05 \times 10^{-5}$
Number of observations	1284		1274	

Significance levels: (***) 0.01.

From Table 2, we note that the effect of the network connectivity covariate IPM on observed raw case incidences is statistically significant at a 0.01 significance level. Further, as expected, the effect of the covariate is positive. The analysis thus demonstrates a significant role of mobility data in understanding the propagation of infectious diseases. Additionally, the analysis validates the ability of our model to convert complex mobility data into simple measures that can be used to study the diffusion of infections via the mobility network.

#### Evaluation of the model performance

**Figure 2:**
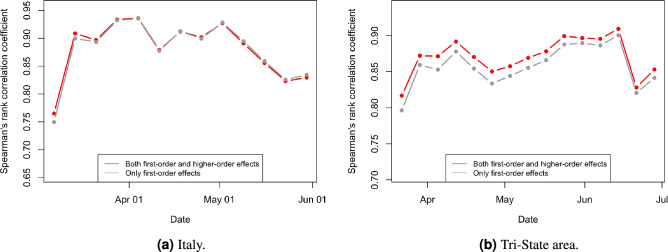
Temporal variation of Spearman’s rank correlation between case incidence and the prevalence metric derived from mobility data and epidemiological inputs.

**Figure 3:**
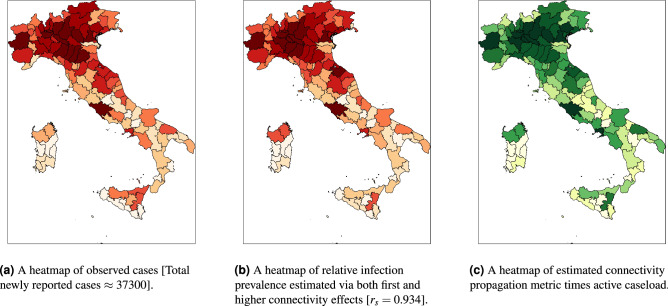
Spatial distributions of estimated infection prevalence and observed new COVID-19 cases in Italy in the week ending 28 March 2020. Top twenty provinces for likely intervention in the order of their rank (by CPM $\times$ active cases): Milano, Bergamo, Brescia, Torino, Roma, Cremona, Napoli, Monza e della Brianza, Padova, Lodi, Pavia, Verona, Treviso, Moden, Piacenza, Bologna, Venezia, Reggio Nell’Emilia, Vicenza, Parma.

**Figure 4:**
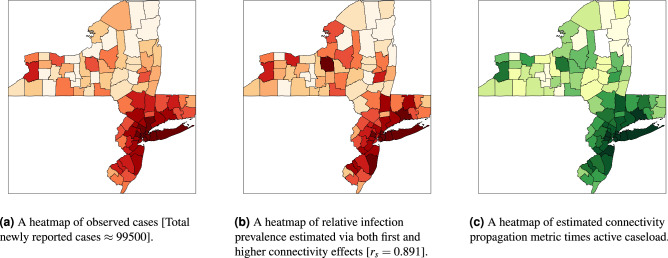
Spatial distributions of estimated infection prevalence and observed new COVID-19 cases in the New York Tri-State Area in the week ending 12 April 2020. Top twenty counties for likely intervention in the order of their rank (by CPM $\times$ active cases): Queens County, Kings County, Nassau County, Suffolk County, Essex County, Bronx County, New Haven County, New York County, Monmouth County, Union County, Westchester County, Bergen County, Middlesex County, Ocean County, Fairfield County, Hudson County, Dutchess County, Camden County, Mercer County, and Richmond County.

We now combine the epidemiological factors (relative infection strength and susceptibility) for a given week with the corresponding ICM (derived from aggregated OD-matrices) to derive zonal-level estimates of the prevalence metric (IPM) using Eq. (4). To validate the model performance, we compute Spearman’s rank correlation coefficient ($r_s$) between the zone-level IPM estimates and observed zonal case counts in the subsequent week for the two case studies. We use Spearman’s correlation because we expect the relationship between the estimated infection prevalence and the observed incidences in the subsequent week to be monotonic but not necessarily linear. Figure 2a,b show the temporal variation of $r_s$ in Italy and the Tri-State area, respectively, over their corresponding study periods. Additionally, Figs. 3b and 4b illustrate the zone-level distribution of the IPM in Italy and the Tri-State area, respectively, in the corresponding week when the highest number new infections were recorded in the whole region. We note that these distributions compare well with the spatial distribution of raw case incidences in that week as shown in Figs. 3a and 4a. Full results; for all weeks in the study period; are attached in the Appendix.

Figure 2a illustrates that as we approach the peak of the first wave of COVID-19 infections in Italy, the value of $r_s$ increases from about 0.77 to 0.94 and then eventually dips to about 0.82 by the end of the infection wave. We expect the actual correlations to be even higher as many COVID-19 case incidences might be missing non-randomly (that is, conditional on local factors) from the recorded data. Such non-random patterns of missingness can occur because rates of case ascertainment may vary substantially across locations depending upon the testing efforts by local officials. A previous study^40^ estimated the ratio of confirmed to actual infections in Italy during the first wave to be only around 10%. Another study^38^ suggested the use of hospitalisation counts as a better indicator for actual cases. However, province-level time-series data on hospitalisations in Italy are not available publicly.

We note from this Fig. 2a that the prevalence metric best predicts future case incidences during the peak of the infection wave (March 20 to April 10) when case counts in the entire system are quite high. A previous study^24^ suggests that the underlying contribution of mobility is quite substantial during the peak infection period. Thus, mobility primarily steers the distribution of raw cases in this period. Moving further along the $r_s$ curve, we note a dip in $r_s$ values, which is again followed by an increase to another local maximum. Note that this period is marked by a full lockdown in Italy, that is, uniform restrictions across all zones (provinces). During this period, the role of mobility continues to decline, while the local strength of the disease (proxied via $R_i$) in each zone becomes a more dominant driver of future case incidences in that zone. The combination of the two effects delivers an approximately U-shaped $r_s$ curve in this period. Interestingly, the $r_s$ values show a monotonic decline through the month of May, which coincides with the period when the lockdown began to ease but intra-zonal travel was still restricted. As noted by a previous study^41^, the local stringency and effectiveness of intervention measures (driven by case counts) primarily determine the distribution of raw case incidence in this period. While such unobserved factors may be partly proxied by $R_i$, the combined role of mobility and active case logs declines continuously over this period.

From Fig. 2b, we note similar patterns in the Tri-state area until the culmination of the full lockdown period (that is, May 15). The estimated $r_s$ first increases from 0.82 to a local maximum value of 0.89 when the infections in the area peak. The local maxima is followed by a U-shaped curve with a local minimum of 0.85. However, unlike the $r_s$ curve for Italy, the $r_s$ values continue to increase monotonically as a phased reopening plan came into force. We note that contrary to the Italian case, the intra-zonal movement was not restricted. Thus mobility seems to once again predominantly govern the spatial distribution of infections in the area. The $r_s$ estimates continue to increase till it reaches another local maximum level of 0.91, beyond which it declines sharply to a level of 0.85 when the overall infections in the area become too low.

#### Understanding the role of higher-order effects

We compute a modified prevalence metric $\tilde{\text {IPM}}$ by considering only the direct transmission path between two nodes, or in other words, only the first-order connectivity effects, using $\tilde{\text {IPM}} = (\mathbf{{A}}\times \mathbf{{R}}) \odot \mathbf{{S}}$. Figure 2a,b plot the Spearman’s rank correlation between the $\tilde{\text {IPM}}$ metric and observed cases in the subsequent week. Figure 2a indicates that the correlations of prevalence metric $\text {IPM}$ are higher than the modified prevalence metric $\tilde{\text {IPM}}$ during the first half of the infection wave in Italy, where mobility has the maximum utility in predicting future cases. Interestingly, in the Tri-State area (see Fig. 2b), the prevalence metric $\text {IPM}$ completely outperforms the modified prevalence metric $\tilde{\text {IPM}}$, as mobility played a substantial role in determining the spread of infections over most of the study period. These findings reinforce the significance of higher-order connectivity in the diffusion of infections through mobility networks. However, the extent of gains due to the inclusion of higher-order effects depends on the data-generating process and can be more apparent in some cases.

#### Informing managerial measures

Figures 3c and 4c show the spatial distribution of the zone-level CPM values (estimated using Eq. (5)) times their active caseload (proxied by cumulative case counts) in the chosen week. As described in the second section, for a given zone, this value signifies the infection effects transmitted in the entire system due to the active infections in that zone. Based on these figures, we identify the top twenty zones to be prioritised for any likely intervention. We enlist these zones in the caption of Figs. 3 and 4.

## Discussion

This study presents a simple compact model of the potential transmission of an infectious disease through transportation networks. It uses readily available aggregate mobility data along with corresponding data on disease incidences as a proxy for unobserved epidemiological factors to estimate the evolution of the disease in the following period. The model formulation provides three important quantities that encapsulate potential disease propagation dynamics in the entire network – the inverse connectivity matrix (ICM), the relative infection prevalence metric (IPM), and the connectivity propagation metric (CPM). The ICM is essentially derived from an inverted origin-destination (OD) travel flow matrix and the cells of this matrix measure successive interaction effects from first and higher-order connectivity between any zone *i* and all other zones. The IPM, obtained from the row sums of ICM, captures the time-varying relative spatial distribution of infections in large geographical areas. Further, the column sums of ICM are used to construct estimates of location-specific CPM. The CPM of a location quantifies the total infection effects propagated in the entire system due to an infection in the location. The uniqueness of the proposed model stems from its ability to capture both first-order and higher-order interactions between different locations in the transportation network. In other words, the model captures the diffusion of infections between two nodes in the network via all possible paths. We find that both first-order and higher-order connectivity effects are significant determinants of the potential diffusion of infections through mobility networks, but the relative importance of higher-order effects will likely vary by context.

We apply the proposed metrics to examine the first wave of COVID-19 infections at two different geographical scales: (1) in Italy at the province level, and, (2) in the New York Tri-State Area at the county level. In both cases, we find that our estimates of zonal (province-level and county-level, respectively) infection prevalence metric (IPM) are highly correlated with the corresponding raw case counts in the subsequent period (Spearman’s rank correlation coefficient between 0.76 and 0.93). Thus, our study reinforces the importance of aggregate mobility data to study the spread of infectious diseases and pandemics. Note that the role of our infection prevalence metric is limited to predicting the relative strength of outbreaks across different geographical areas as opposed to predicting the actual case incidence. As a part of future work, we aim to work with epidemiologists to incorporate the proposed inverted OD formulation in a traditional epidemiological model (for instance, the SEIR model) to predict actual case counts. Another important avenue for future research is to adapt the formulation for the inverse problem of identifying the source of outbreaks in complex networks.

The CPM estimates of an area could be particularly useful for public health authorities during the early stages of an outbreak or a resurgence of an infectious disease to identify target areas and the required magnitude of intervention. Moreover, the IPM estimates of an area could be employed as a performance metric to evaluate the effectiveness of intervention measures introduced in that area by benchmarking the predictions against real-time case counts.

## Supplementary Information

Supplementary Information.

## Data Availability

The Italian mobility dataset is obtained from the Humanitarian Data Exchange website: https://data.humdata.org/dataset/covid-19-mobility-italy. The mobility data for the Tri-State Area is available via GitHub at https://github.com/GeoDS/COVID19USFlows. COVID-19 epidemiological data for Italy is procured from a public repository maintained at https://github.com/pcm-dpc/COVID-19. Similar caseload data for Tri-State area counties is obtained from https://usafacts.org/articles/detailed-methodology-covid-19-data/. Population estimates for Italian provinces and Tri-State area counties are extracted from http://citypopulation.de/en/italy/admin/ and https://www.census.gov/data/datasets/time-series/demo/popest/2010s-counties-total.html, respectively. The authors are happy to provide the assembled data and code upon request.
