# Supplementary material for: Modelling the propagation of infectious disease via transportation networks
Source: Sci Rep. 2022 Nov 29;12:20572. doi: 10.1038/s41598-022-24866-3 (PMC9707165; doi:10.1038/s41598-022-24866-3)
Supplement: Supplementary file 1 — Supplementary Information. [file 41598_2022_24866_MOESM1_ESM.pdf]

Modelling the propagation of infectious disease via transportation networks — Supplementary Information File

Anupriya[1,+], Prateek Bansal[2,+], and Daniel J. Graham[1,*,+]

[1]Transport Strategy Centre, Department of Civil and Environmental Engineering, Imperial College London, London SW7 2AZ, UK
[2]Department of Civil and Environmental Engineering, National University of Singapore, Singapore 119077
[*]d.j.graham@imperial.ac.uk
[+]These authors contributed equally to this work.

ABSTRACT

This supporting information file supplements the model and results section in the main paper.

Steps to implement the proposed model

Algorithm 1: THE INVERSE CONNECTIVITY MATRIX

Input : OD matrix
Output : Inverse connectivity matrix (ICM)

1 Assemble the $n \times n$ OD matrix with elements x_{ij} representing trips from origin i to destination j; $i, j = 1, 2, \dots, n$.
2 Construct the $n \times 1$ vectors **O** and **D** with elements $O_i = \sum_{j=1}^{n} x_{ij}$ and $D_i = \sum_{j=1}^{n} x_{ji}$, respectively.
3 Estimate the $n \times 1$ zonal scale vector **t** with elements $t_i = (O_i + D_i)/2$.
4 Derive the $n \times 1$ total propagation potential vector **p** with elements $p_i = t_i + D_i$.
5 Generate the $n \times n$ **A** matrix with elements $a_{ij} = x_{ji}/p_j$.
6 Invert the **A** matrix to obtain the $n \times n$ inverse connectivity matrix (ICM) with elements b_{ij}.

Algorithm 2: THE RELATIVE INFECTION PREVALENCE METRIC

Input : Inverse connectivity matrix (ICM), **t** vector, Case log, Population estimates, Population immunity estimates
Output : Relative infection prevalence metric (IPM)

1 Construct a $n \times 1$ vector representing the relative zonal strength of the infectious disease **R**, with elements

$$R_i = \left(\frac{\text{Active cases in zone } i}{\sum_{i=1}^{n} \text{Active cases in zone } i} \Bigg/ \frac{\text{Population of zone } i}{\sum_{i=1}^{n} \text{Population of zone } i} \right) \times t_i.$$

2 Generate the $n \times 1$ zonal susceptibility vector **S** with elements
$S_i =$ Population of zone $i -$ Number of immune individuals in zone i.
3 Derive the $n \times 1$ vector of the relative infection prevalence metric $\text{IPM} = (\text{ICM} \times \mathbf{R}) \odot \mathbf{S}$.

Algorithm 3: THE CONNECTIVITY PROPAGATION METRIC

Input : ICM, Case log, **S** vector, **t** vector
Output : Connectivity Propagation Metric (CPM)

1 Derive the $n \times 1$ vector connectivity propagation metric (CPM) with elements $\text{CPM}_i = t_i \sum_{j=1}^{n} b_{ji} S_j$.
2 Multiply each element of the CPM vector with the active cases in the corresponding zone.
3 Arrange the resulting metric from Step 2 in ascending order to identify top zones for intervention.

Full Results

Figures 1 to 13 show the spatial distributions of estimated relative infection prevalence and observed weekly COVID-19 case incidences in Italy during the associated study period. Similarly, Figures 14 to 28 show the spatial distributions of estimated relative infection prevalence and observed weekly COVID-19 case incidences in the New York Tri-State Area during the associated study period.

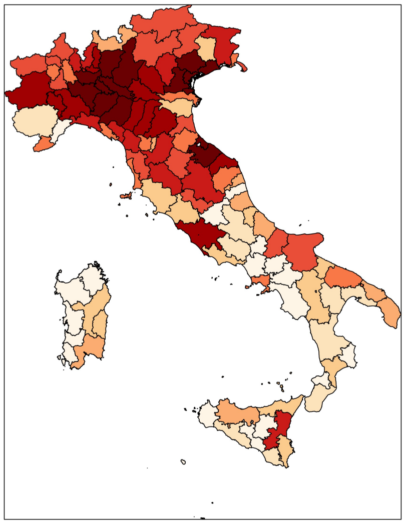

(a) A heatmap of observed cases [Total newly reported cases ≈ 4600].

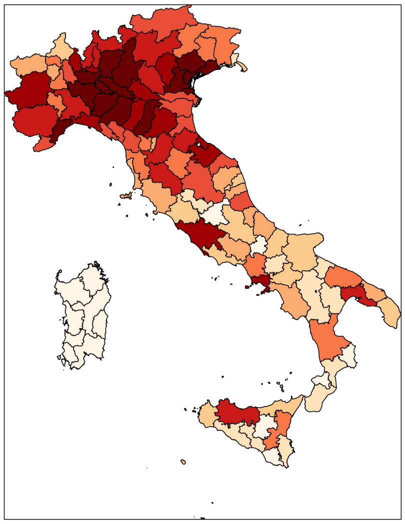

(b) A heatmap of relative infection prevalence estimated via both first and higher connectivity effects [$r_s = 0.765$].

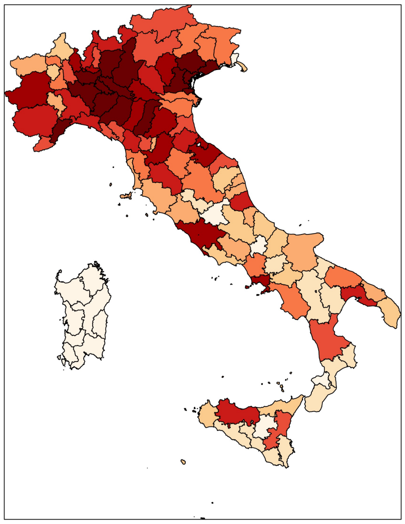

(c) A heatmap of relative infection prevalence estimated via first-order connectivity effects [$r_s = 0.749$].

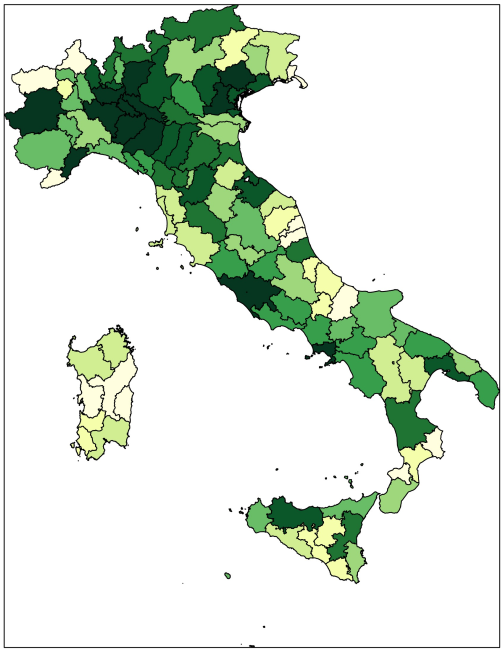

(d) A heatmap of estimated connectivity propagation metric times active caseload.

Name of the province	Rank (by CPM× active cases)
Lodi	1
Bergamo	2
Cremona	3
Padova	4
Piacenza	5
Milano	6
Pavia	7
Napoli	8
Treviso	9
Parma	10
Torino	11
Savona	12
Roma	13
Brescia	14
Modena	15
Venezia	16
Monza e della Brianza	17
Rimini	18
Varese	19
Pesaro E Urbino	20

(e) List of top twenty provinces for likely intervention.

Figure 1. Spatial distributions of estimated relative infection prevalence and observed new COVID-19 cases in the week ending 7 March 2020.

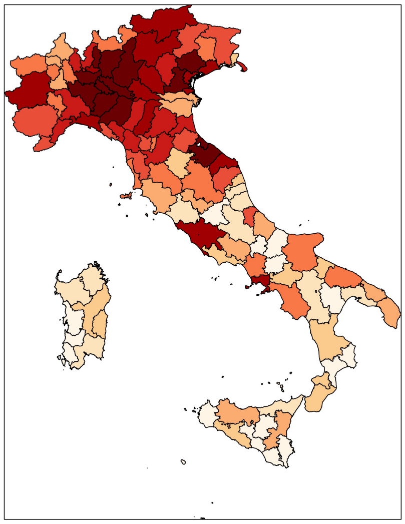

(a) A heatmap of observed cases [Total newly reported cases $\approx$ 14600].

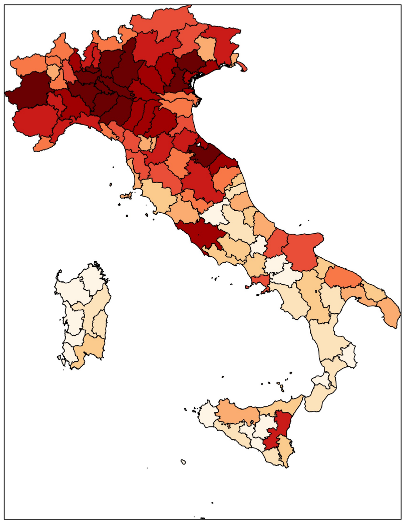

(b) A heatmap of relative infection prevalence estimated via both first and higher connectivity effects [$r_s = 0.909$].

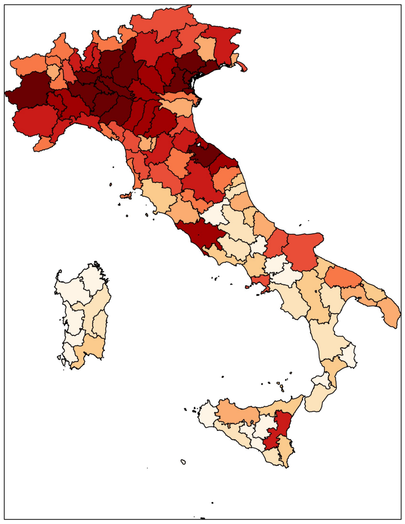

(c) A heatmap of relative infection prevalence estimated via first-order connectivity effects [$r_s = 0.899$].

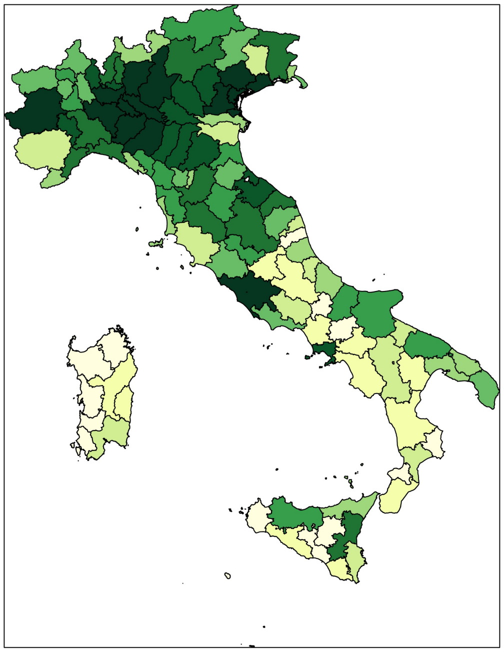

(d) A heatmap of estimated connectivity propagation metric times active caseload.

Name of the province	Rank (by CPM$\times$ active cases)
Bergamo	1
Lodi	2
Milano	3
Brescia	4
Cremona	5
Piacenza	6
Pavia	7
Roma	8
Padova	9
Parma	10
Torino	11
Treviso	12
Venezia	13
Monza e della Brianza	14
Pesaro E Urbino	15
Modena	16
Verona	17
Rimini	18
Bologna	19
Napoli	20

(e) List of top twenty provinces for likely intervention.

Figure 2. Spatial distributions of estimated relative infection prevalence and observed new COVID-19 cases in the week ending 14 March 2020.

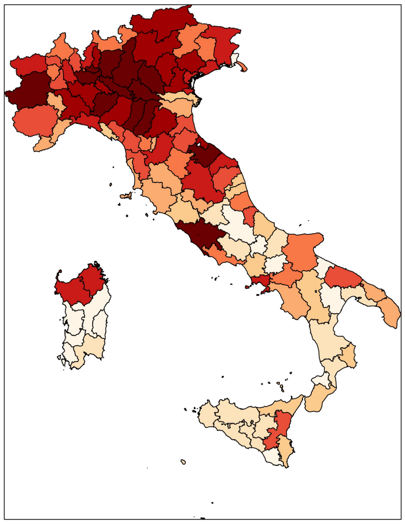

(a) A heatmap of observed cases [Total newly reported cases ≈ 31900].

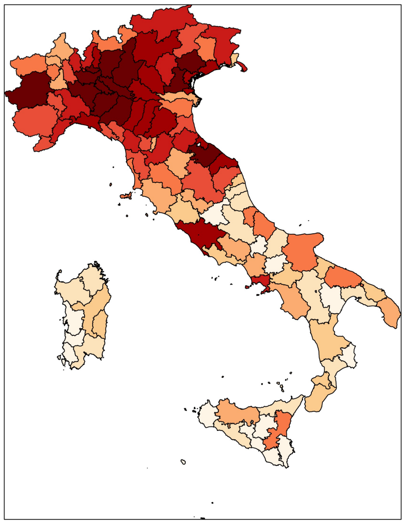

(b) A heatmap of relative infection prevalence estimated via both first and higher connectivity effects [$r_s = 0.897$].

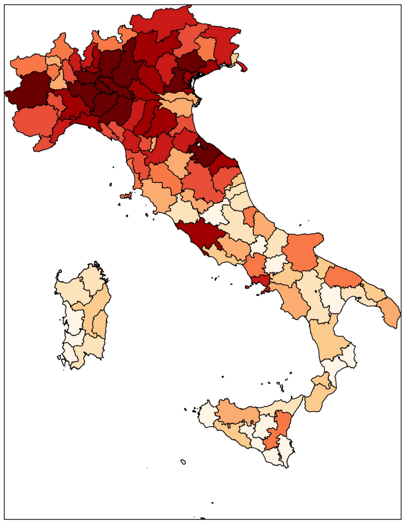

(c) A heatmap of relative infection prevalence estimated via first-order connectivity effects [$r_s = 0.893$].

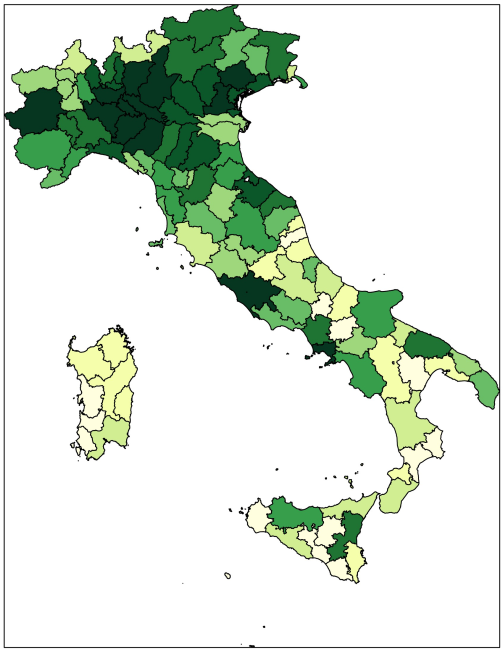

(d) A heatmap of estimated connectivity propagation metric times active caseload.

Name of the province	Rank (by CPM× active cases)
Milano	1
Bergamo	2
Brescia	3
Cremona	4
Roma	5
Lodi	6
Torino	7
Padova	8
Pavia	9
Napoli	10
Piacenza	11
Treviso	12
Parma	13
Monza e della Brianza	14
Verona	15
Venezia	16
Pesaro E Urbino	17
Modena	18
Genova	19
Lecco	20

(e) List of top twenty provinces for likely intervention.

Figure 3. Spatial distributions of estimated relative infection prevalence and observed new COVID-19 cases in the week ending 21 March 2020.

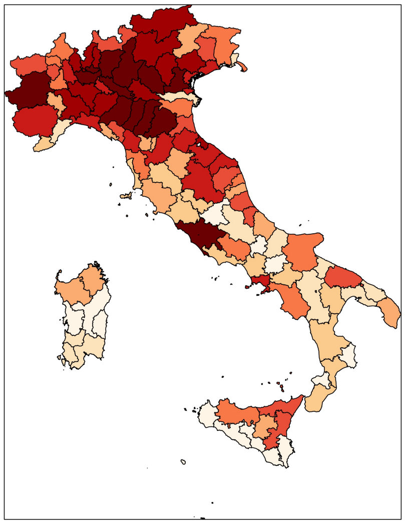

(a) A heatmap of observed cases [Total newly reported cases $\approx$ 37300].

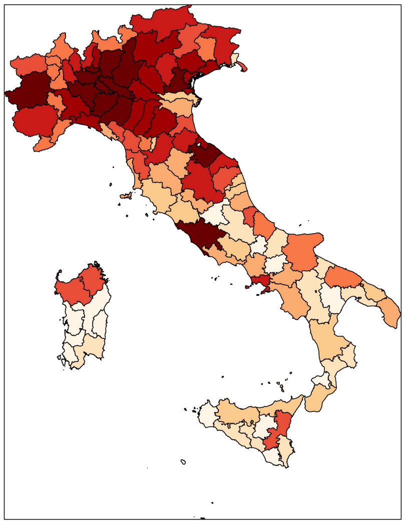

(b) A heatmap of relative infection prevalence estimated via both first and higher connectivity effects [$r_s = 0.934$].

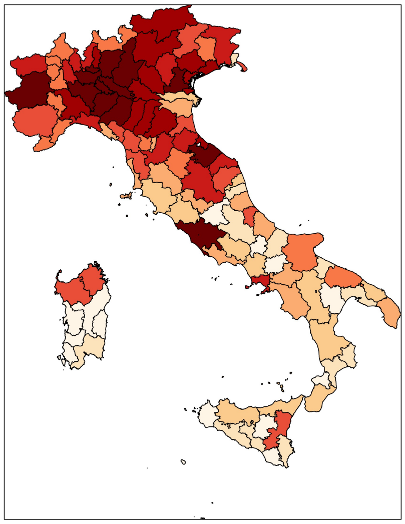

(c) A heatmap of relative infection prevalence estimated via first-order connectivity effects [$r_s = 0.932$].

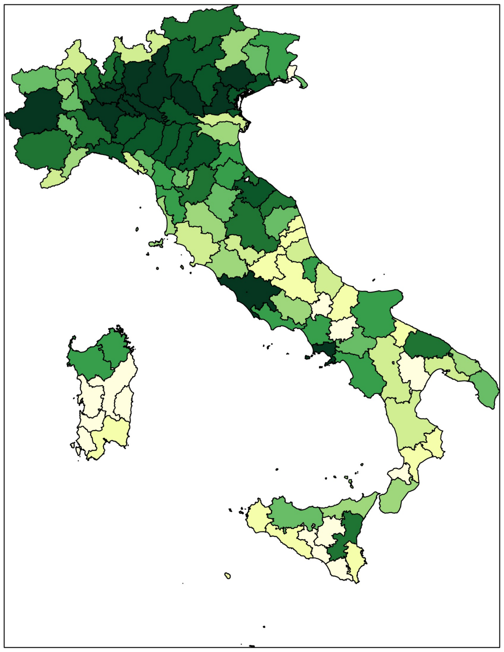

(d) A heatmap of estimated connectivity propagation metric times active caseload.

Name of the province	Rank (by CPM$\times$ active cases)
Milano	1
Bergamo	2
Brescia	3
Torino	4
Roma	5
Cremona	6
Napoli	7
Monza e della Brianza	8
Padova	9
Lodi	10
Pavia	11
Verona	12
Treviso	13
Modena	14
Piacenza	15
Bologna	16
Venezia	17
Reggio Nell'Emilia	18
Vicenza	19
Parma	20

(e) List of top twenty provinces for likely intervention.

Figure 4. Spatial distributions of estimated relative infection prevalence and observed new COVID-19 cases in the week ending 28 March 2020.

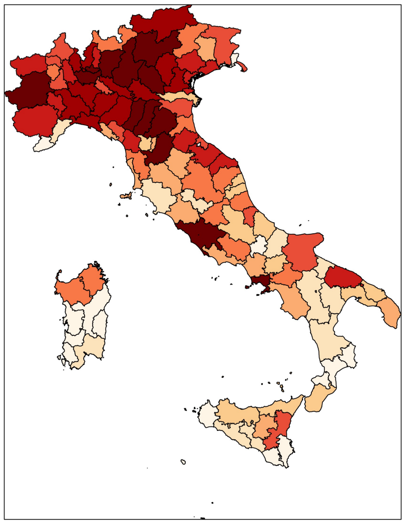

(a) A heatmap of observed cases [Total newly reported cases $\approx$ 31200].

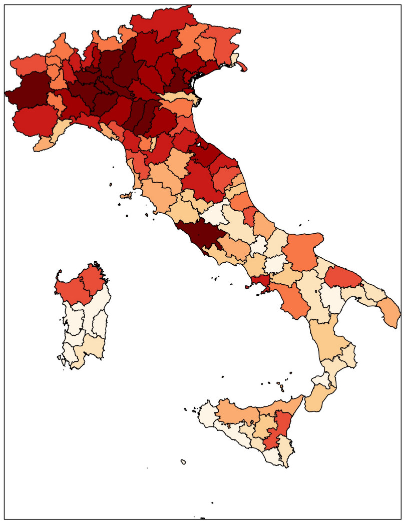

(b) A heatmap of relative infection prevalence estimated via both first and higher connectivity effects [$r_s = 0.936$].

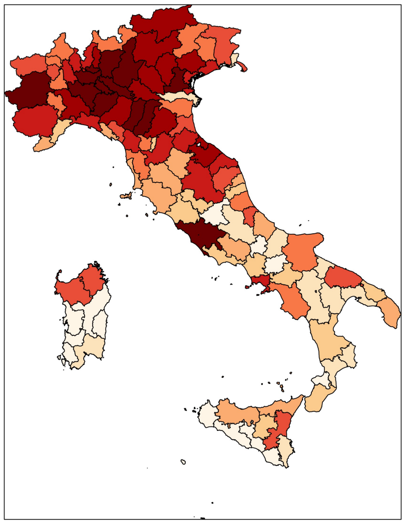

(c) A heatmap of relative infection prevalence estimated via first-order connectivity effects [$r_s = 0.935$].

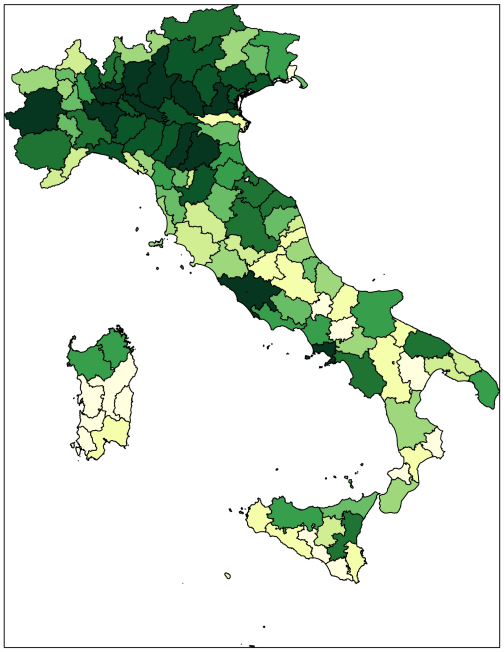

(d) A heatmap of estimated connectivity propagation metric times active caseload.

Name of the province	Rank (by CPM× active cases)
Milano	1
Brescia	2
Bergamo	3
Torino	4
Roma	5
Napoli	6
Monza e della Brianza	7
Padova	8
Cremona	9
Verona	10
Bologna	11
Modena	12
Pavia	13
Treviso	14
Reggio Nell'Emilia	15
Vicenza	16
Lodi	17
Venezia	18
Trento	19
Parma	20

(e) List of top twenty provinces for likely intervention.

Figure 5. Spatial distributions of estimated relative infection prevalence and observed new COVID-19 cases in the week ending 4 April 2020.

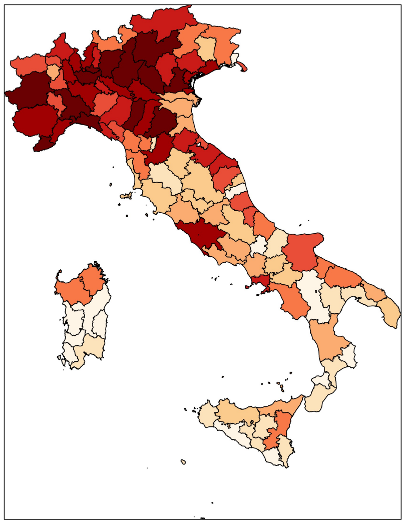

(a) A heatmap of observed cases [Total newly reported cases ≈ 29000].

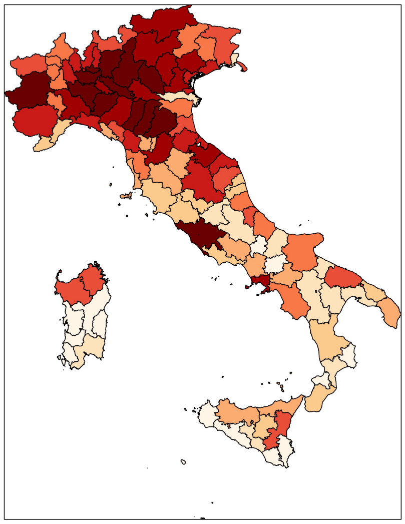

(b) A heatmap of relative infection prevalence estimated via both first and higher connectivity effects [$r_s = 0.879$].

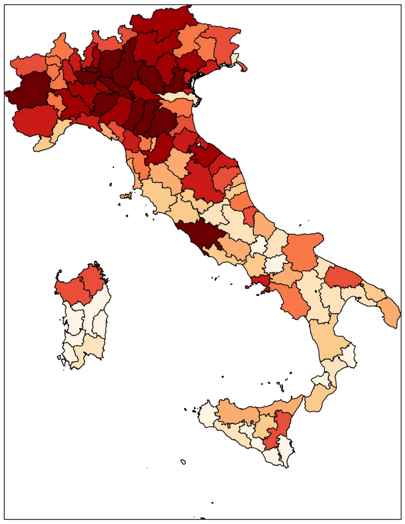

(c) A heatmap of relative infection prevalence estimated via first-order connectivity effects [$r_s = 0.878$].

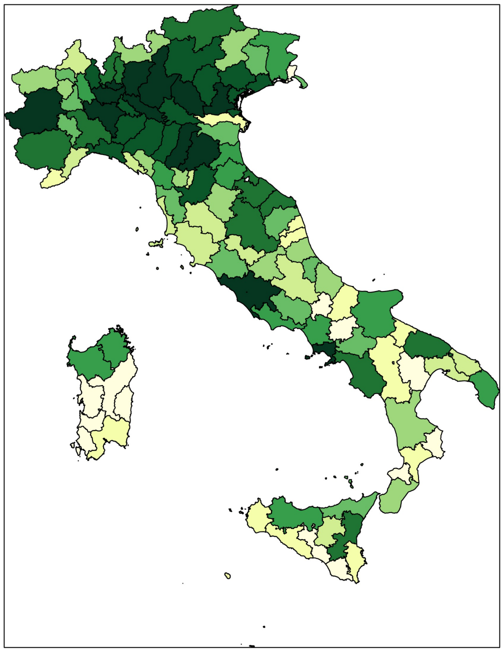

(d) A heatmap of estimated connectivity propagation metric times active caseload.

Name of the province	Rank (by CPM× active cases)
Milano	1
Torino	2
Brescia	3
Bergamo	4
Roma	5
Napoli	6
Monza e della Brianza	7
Padova	8
Verona	9
Bologna	10
Cremona	11
Modena	12
Pavia	13
Reggio Nell'Emilia	14
Treviso	15
Firenze	16
Vicenza	17
Trento	18
Varese	19
Venezia	20

(e) List of top twenty provinces for likely intervention.

Figure 6. Spatial distributions of estimated relative infection prevalence and observed new COVID-19 cases in the week ending 11 April 2020.

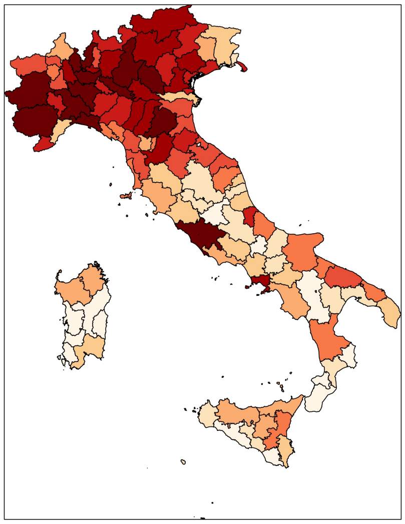

(a) A heatmap of observed cases [Total newly reported cases $\approx$ 23600].

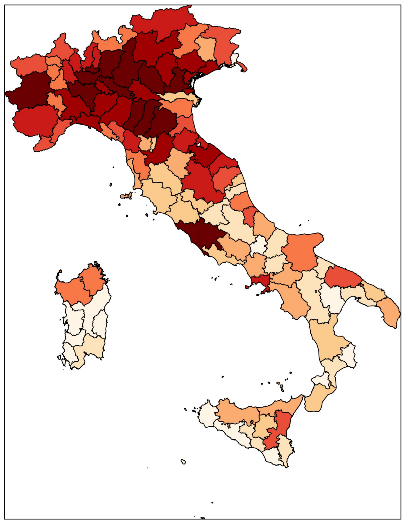

(b) A heatmap of relative infection prevalence estimated via both first and higher connectivity effects [$r_s = 0.912$].

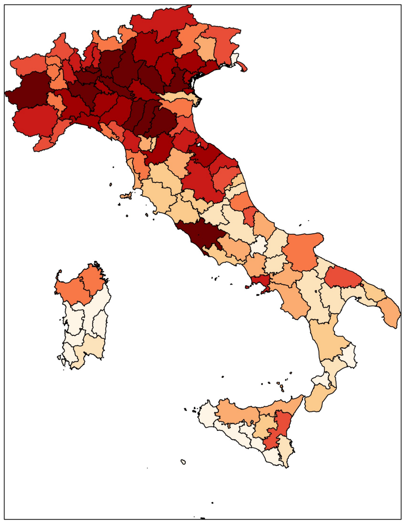

(c) A heatmap of relative infection prevalence estimated via first-order connectivity effects [$r_s = 0.912$].

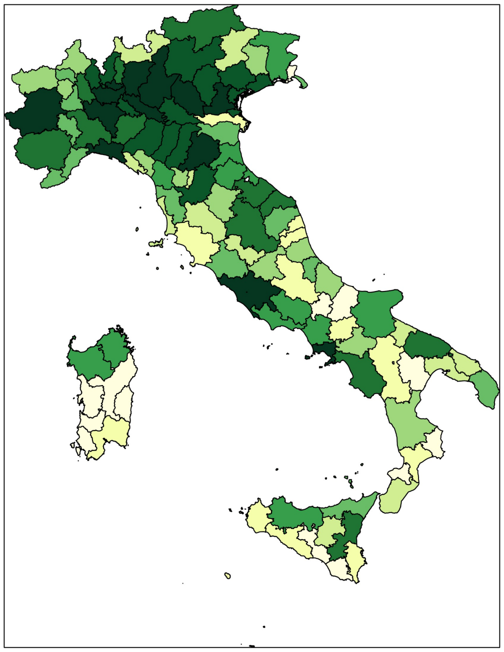

(d) A heatmap of estimated connectivity propagation metric times active caseload.

Name of the province	Rank (by CPM× active cases)
Milano	1
Torino	2
Brescia	3
Roma	4
Bergamo	5
Napoli	6
Monza e della Brianza	7
Verona	8
Padova	9
Bologna	10
Genova	11
Cremona	12
Pavia	13
Modena	14
Reggio Nell'Emilia	15
Firenze	16
Vicenza	17
Treviso	18
Varese	19
Trento	20

(e) List of top twenty provinces for likely intervention.

Figure 7. Spatial distributions of estimated relative infection prevalence and observed new COVID-19 cases in the week ending 18 April 2020.

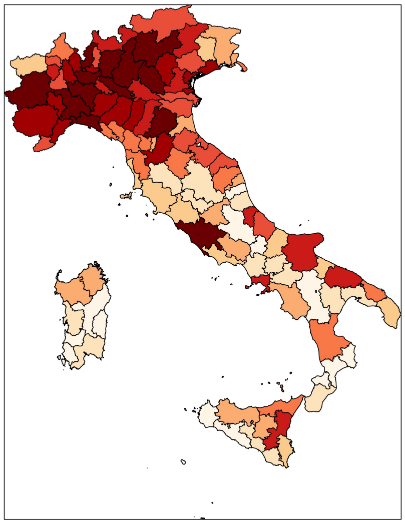

(a) A heatmap of observed cases [Total newly reported cases $\approx$ 19200].

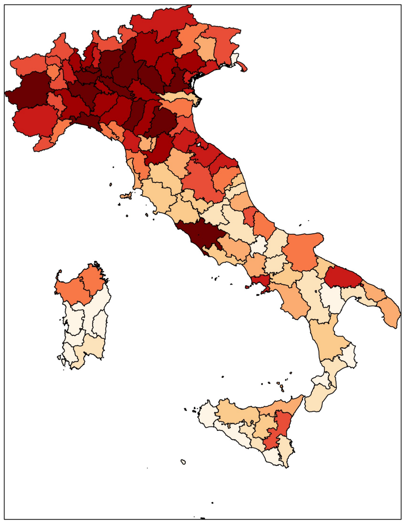

(b) A heatmap of relative infection prevalence estimated via both first and higher connectivity effects [$r_s = 0.902$].

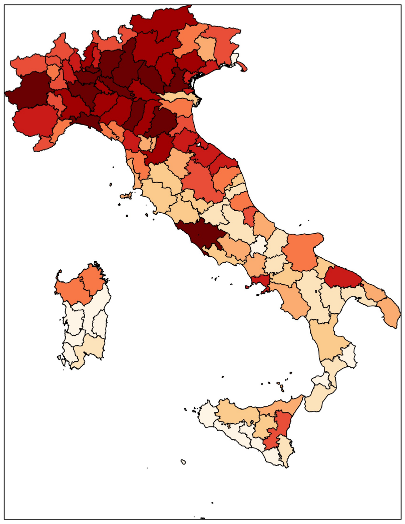

(c) A heatmap of relative infection prevalence estimated via first-order connectivity effects [$r_s = 0.899$].

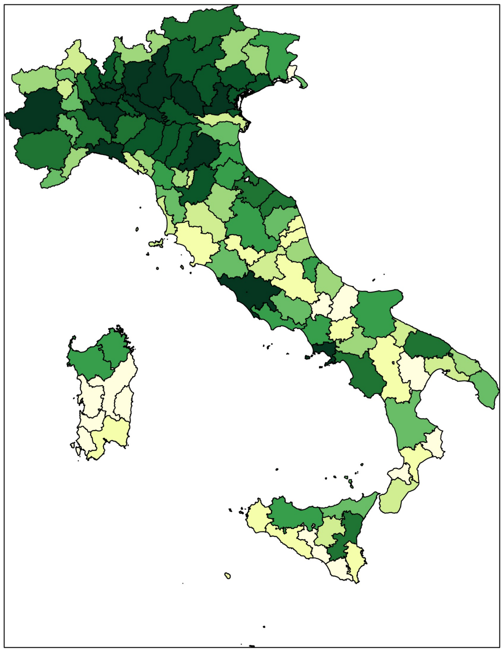

(d) A heatmap of estimated connectivity propagation metric times active caseload.

Name of the province	Rank (by CPM$\times$ active cases)
Milano	1
Torino	2
Roma	3
Brescia	4
Bergamo	5
Napoli	6
Monza e della Brianza	7
Verona	8
Bologna	9
Padova	10
Genova	11
Cremona	12
Pavia	13
Modena	14
Reggio Nell'Emilia	15
Firenze	16
Varese	17
Vicenza	18
Treviso	19
Trento	20

(e) List of top twenty provinces for likely intervention.

Figure 8. Spatial distributions of estimated relative infection prevalence and observed new COVID-19 cases in the week ending 25 April 2020.

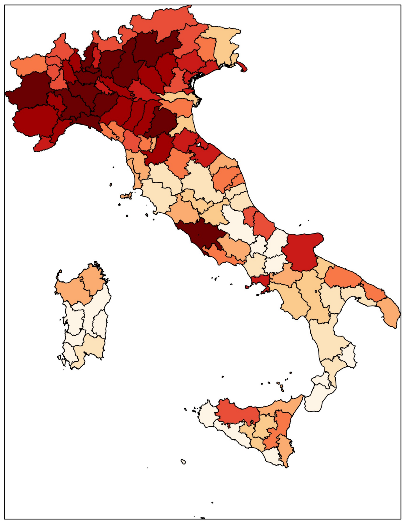

(a) A heatmap of observed cases [Total newly reported cases ≈ 13800].

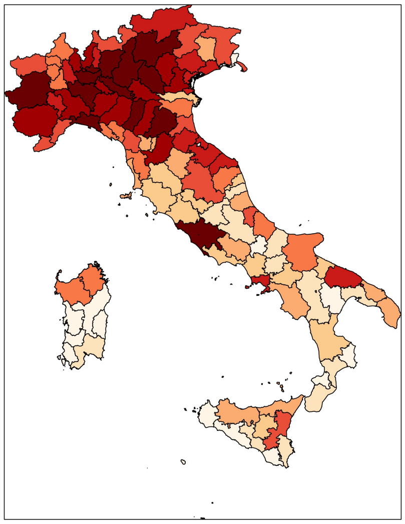

(b) A heatmap of relative infection prevalence estimated via both first and higher connectivity effects [$r_s = 0.927$].

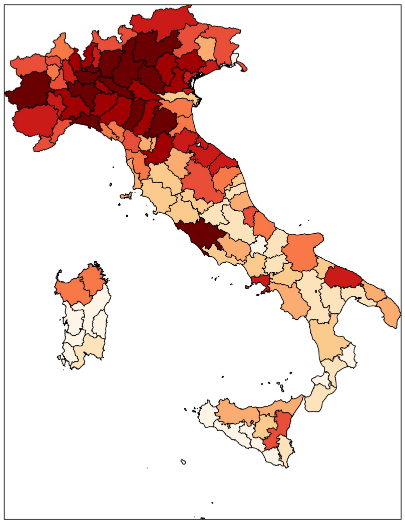

(c) A heatmap of relative infection prevalence estimated via first-order connectivity effects [$r_s = 0.928$].

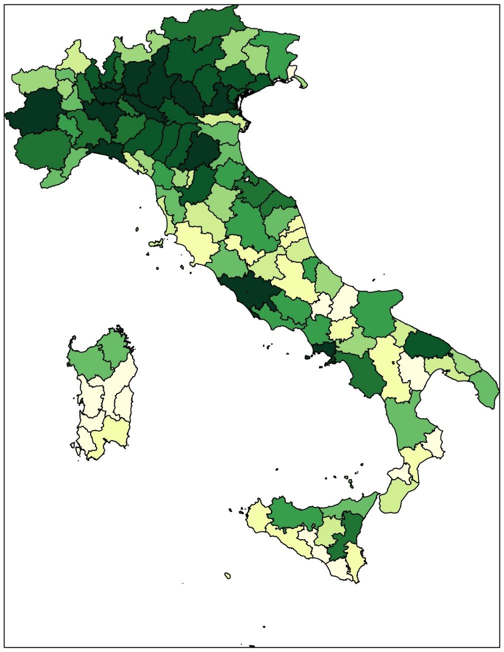

(d) A heatmap of estimated connectivity propagation metric times active caseload.

Name of the province	Rank (by CPM× active cases)
Milano	1
Torino	2
Roma	3
Brescia	4
Bergamo	5
Napoli	6
Monza e della Brianza	7
Verona	8
Bologna	9
Genova	10
Padova	11
Cremona	12
Pavia	13
Reggio Nell'Emilia	14
Varese	15
Firenze	16
Modena	17
Vicenza	18
Trento	19
Treviso	20

(e) List of top twenty provinces for likely intervention.

Figure 9. Spatial distributions of estimated relative infection prevalence and observed new COVID-19 cases in the week ending 2 May 2020.

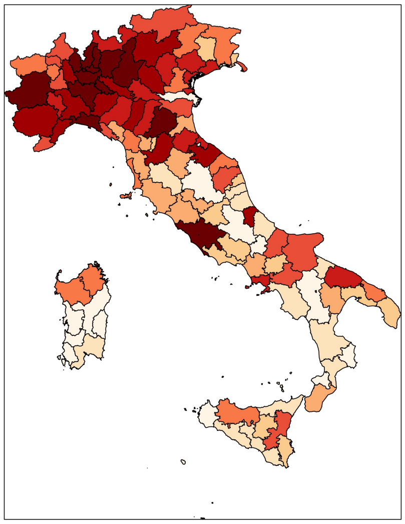

(a) A heatmap of observed cases [Total newly reported cases $\approx$ 8800].

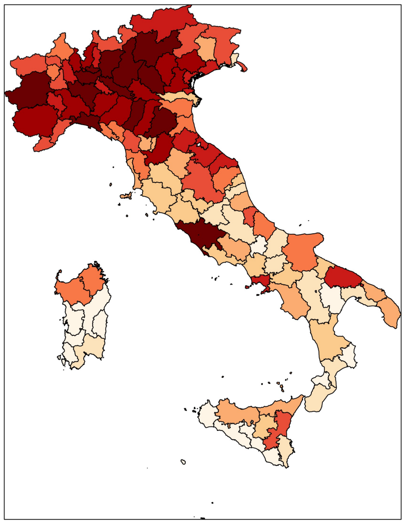

(b) A heatmap of relative infection prevalence estimated via both first and higher connectivity effects [$r_s = 0.891$].

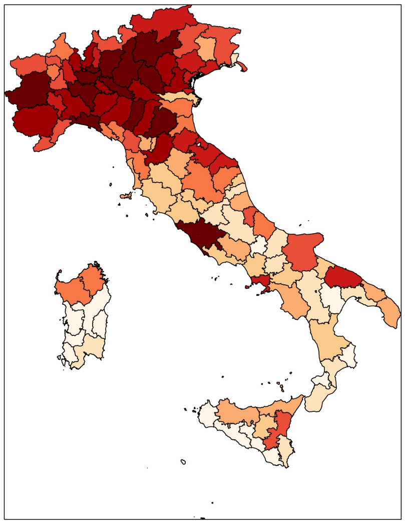

(c) A heatmap of relative infection prevalence estimated via first-order connectivity effects [$r_s = 0.895$].

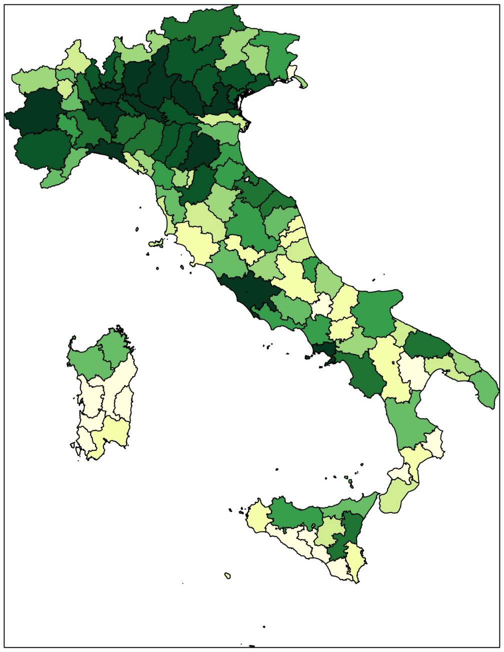

(d) A heatmap of estimated connectivity propagation metric times active caseload.

Name of the province	Rank (by CPM$\times$ active cases)
Milano	1
Torino	2
Roma	3
Brescia	4
Bergamo	5
Napoli	6
Monza e della Brianza	7
Verona	8
Bologna	9
Genova	10
Padova	11
Cremona	12
Pavia	13
Varese	14
Firenze	15
Reggio Nell'Emilia	16
Modena	17
Vicenza	18
Trento	19
Treviso	20

(e) List of top twenty provinces for likely intervention.

Figure 10. Spatial distributions of estimated relative infection prevalence and observed new COVID-19 cases in the week ending 9 May 2020.

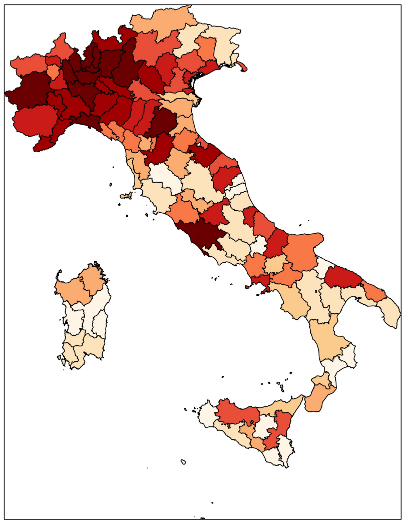

(a) A heatmap of observed cases [Total newly reported cases $\approx$ 6400].

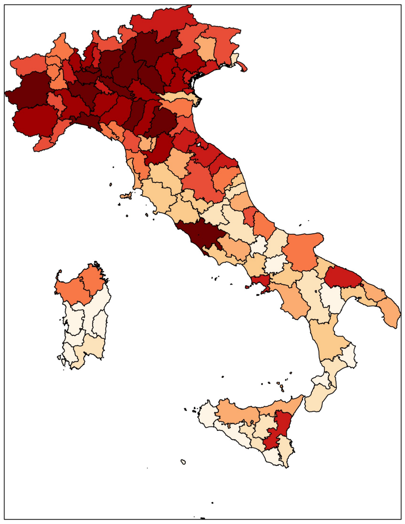

(b) A heatmap of relative infection prevalence estimated via both first and higher connectivity effects [$r_s = 0.855$].

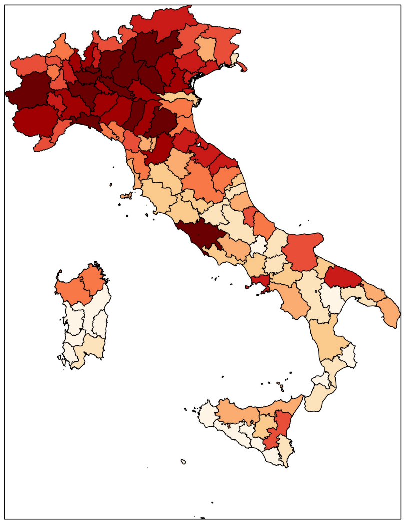

(c) A heatmap of relative infection prevalence estimated via first-order connectivity effects [$r_s = 0.859$].

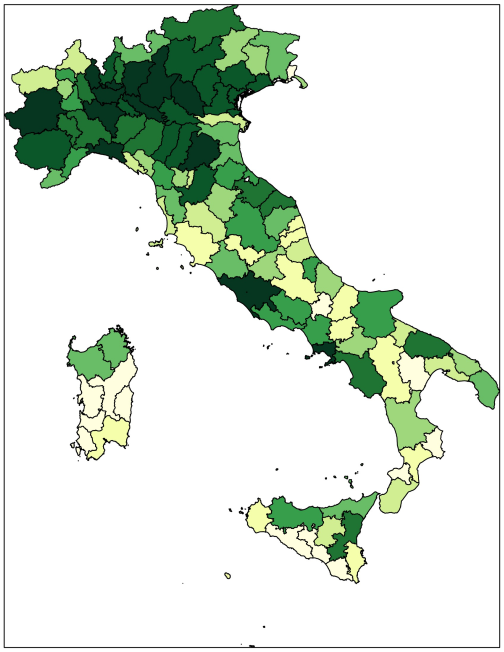

(d) A heatmap of estimated connectivity propagation metric times active caseload.

Name of the province	Rank (by CPM$\times$ active cases)
Milano	1
Torino	2
Roma	3
Brescia	4
Bergamo	5
Napoli	6
Monza e della Brianza	7
Verona	8
Bologna	9
Genova	10
Pavia	11
Cremona	12
Varese	13
Padova	14
Reggio Nell'Emilia	15
Modena	16
Firenze	17
Como	18
Vicenza	19
Trento	20

(e) List of top twenty provinces for likely intervention.

Figure 11. Spatial distributions of estimated relative infection prevalence and observed new COVID-19 cases in the week ending 16 May 2020.

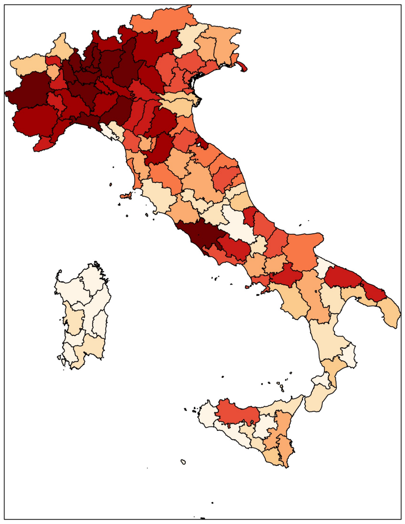

(a) A heatmap of observed cases [Total newly reported cases $\approx$ 4500].

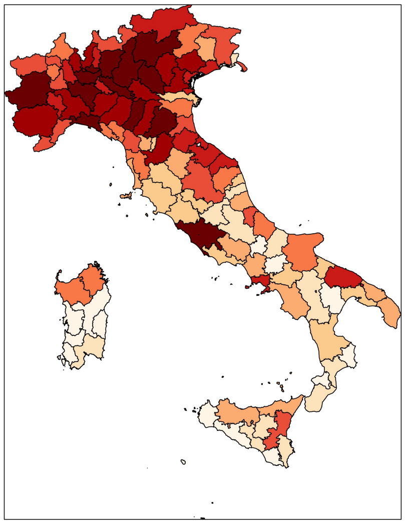

(b) A heatmap of relative infection prevalence estimated via both first and higher connectivity effects [$r_s = 0.823$].

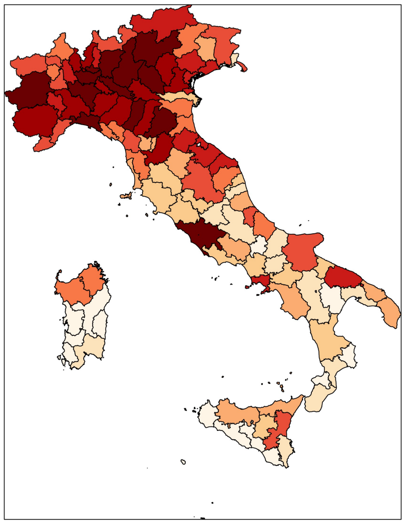

(c) A heatmap of relative infection prevalence estimated via first-order connectivity effects [$r_s = 0.826$].

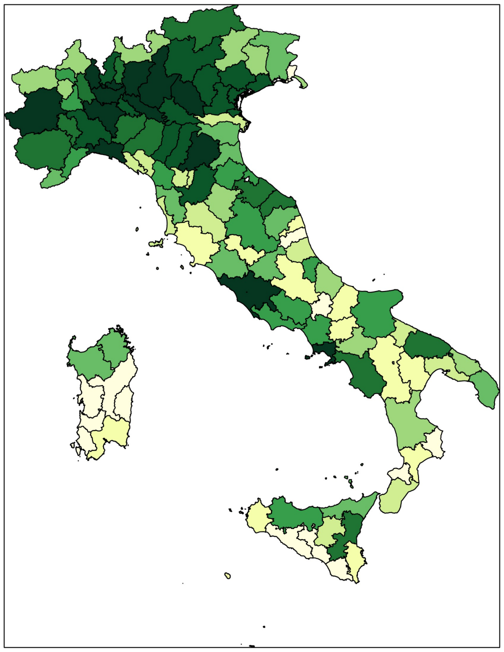

(d) A heatmap of estimated connectivity propagation metric times active caseload.

Name of the province	Rank (by CPM$\times$ active cases)
Milano	1
Torino	2
Roma	3
Brescia	4
Bergamo	5
Napoli	6
Monza e della Brianza	7
Verona	8
Genova	9
Bologna	10
Pavia	11
Cremona	12
Varese	13
Padova	14
Reggio Nell'Emilia	15
Modena	16
Como	17
Firenze	18
Lodi	19
Vicenza	20

(e) List of top twenty provinces for likely intervention.

Figure 12. Spatial distributions of estimated relative infection prevalence and observed new COVID-19 cases in the week ending 23 May 2020.

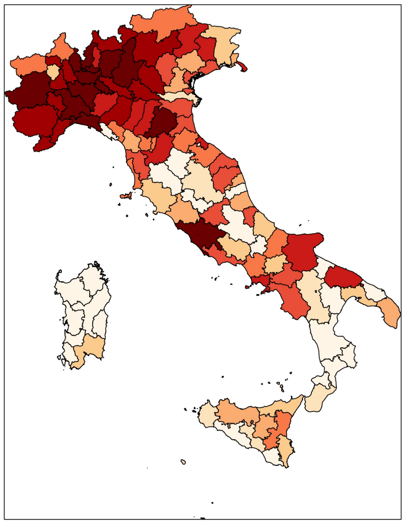

(a) A heatmap of observed cases [Total newly reported cases $\approx$ 3300].

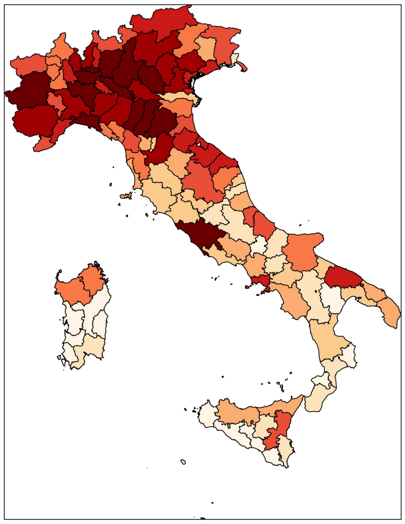

(b) A heatmap of relative infection prevalence estimated via both first and higher connectivity effects [$r_s = 0.830$].

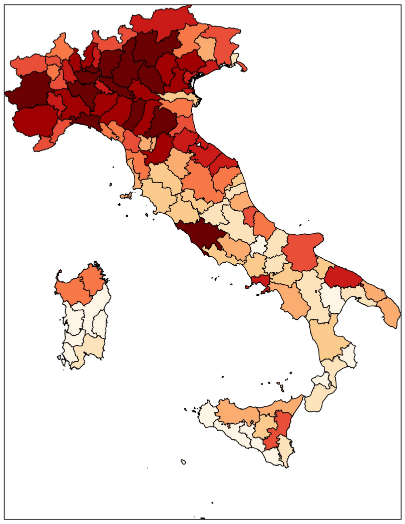

(c) A heatmap of relative infection prevalence estimated via first-order connectivity effects [$r_s = 0.834$].

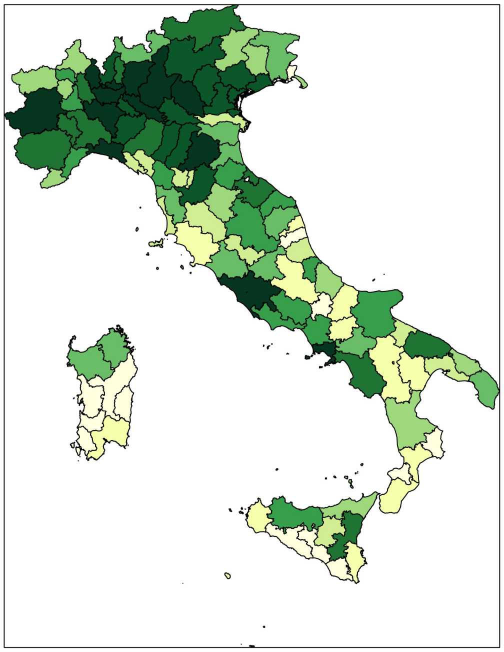

(d) A heatmap of estimated connectivity propagation metric times active caseload.

Name of the province	Rank (by CPM× active cases)
Milano	1
Torino	2
Roma	3
Brescia	4
Bergamo	5
Monza e della Brianza	6
Napoli	7
Pavia	8
Verona	9
Genova	10
Bologna	11
Varese	12
Cremona	13
Padova	14
Como	15
Reggio Nell'Emilia	16
Lodi	17
Modena	18
Firenze	19
Vicenza	20

(e) List of top twenty provinces for likely intervention.

Figure 13. Spatial distributions of estimated relative infection prevalence and observed new COVID-19 cases in the week ending 30 May 2020.

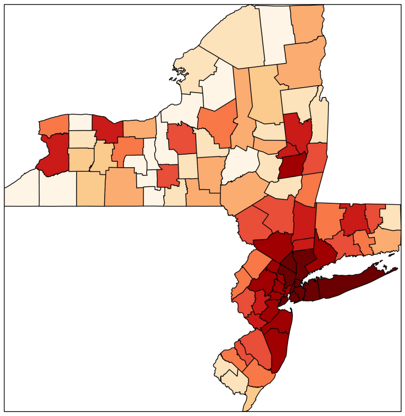

(a) A heatmap of observed cases [Total newly reported cases $\approx$ 17000].

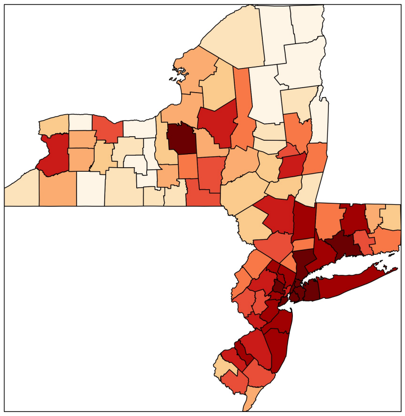

(b) A heatmap of relative infection prevalence estimated via both first and higher connectivity effects [$r_s = 0.817$].

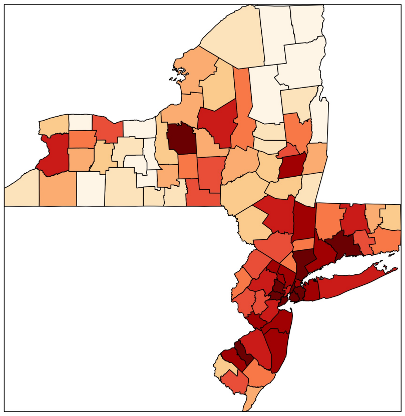

(c) A heatmap of relative infection prevalence estimated via first-order connectivity effects [$r_s = 0.796$].

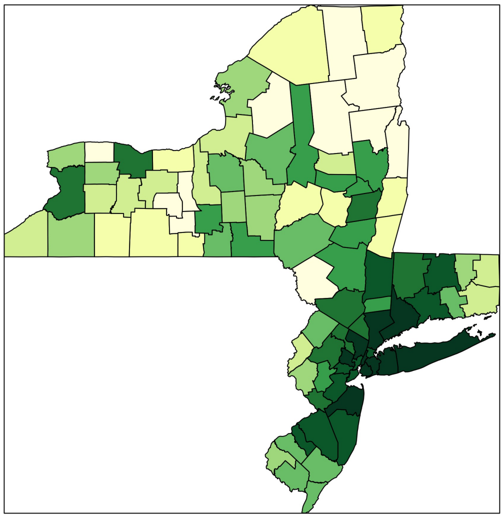

(d) A heatmap of estimated connectivity propagation metric times active caseload.

Name of the county	Rank (by CPM$\times$ active cases)
Queens County	1
Kings County	2
Westchester County	3
Nassau County	4
New York County	5
Suffolk County	6
Monmouth County	7
Essex County	8
Bergen County	9
Fairfield County	10
New Haven County	11
Middlesex County	12
Bronx County	13
Dutchess County	14
Union County	15
Hudson County	16
Camden County	17
Richmond County	18
Burlington County	19
Ocean County	20

(e) List of top twenty counties for likely intervention.

Figure 14. Spatial distributions of estimated relative infection prevalence and observed new COVID-19 cases in the week ending 22 March 2020.

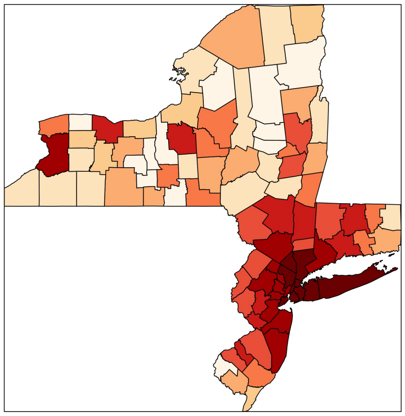

(a) A heatmap of observed cases [Total newly reported cases ≈ 53800].

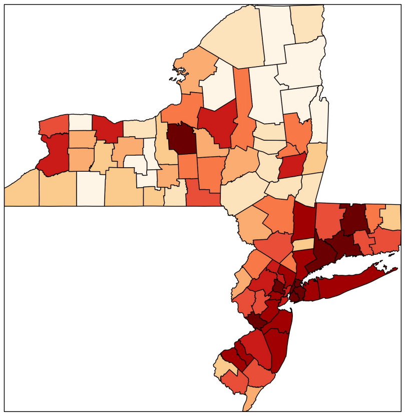

(b) A heatmap of relative infection prevalence estimated via both first and higher connectivity effects [$r_s = 0.872$].

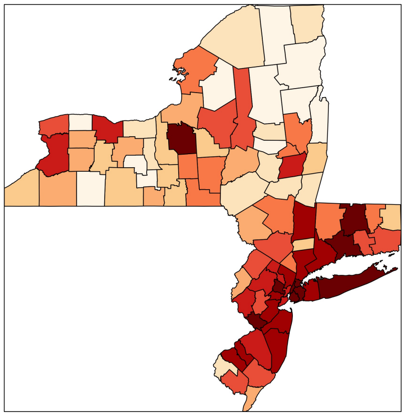

(c) A heatmap of relative infection prevalence estimated via first-order connectivity effects [$r_s = 0.859$].

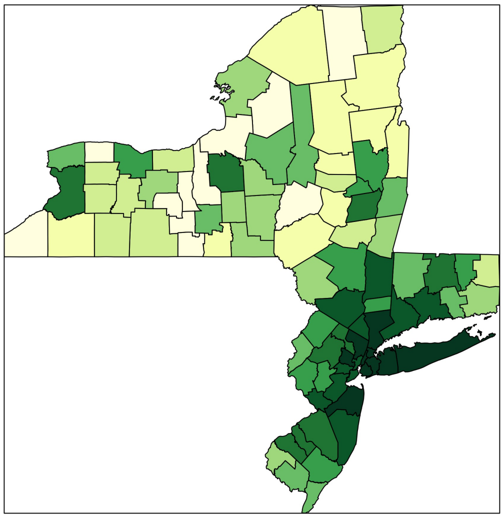

(d) A heatmap of estimated connectivity propagation metric times active caseload.

Name of the county	Rank (by CPM× active cases)
Queens County	1
Kings County	2
Nassau County	3
New York County	4
Suffolk County	5
Bronx County	6
Westchester County	7
Monmouth County	8
Essex County	9
Bergen County	10
Fairfield County	11
Union County	12
Middlesex County	13
Richmond County	14
Ocean County	15
New Haven County	16
Dutchess County	17
Mercer County	18
Hudson County	19
Rockland County	20

(e) List of top twenty counties for likely intervention.

Figure 15. Spatial distributions of estimated relative infection prevalence and observed new COVID-19 cases in the week ending 29 March 2020.

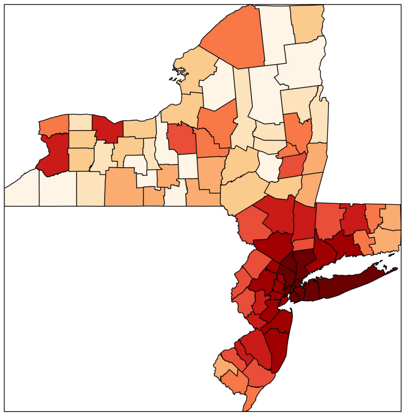

(a) A heatmap of observed cases [Total newly reported cases $\approx$ 89800].

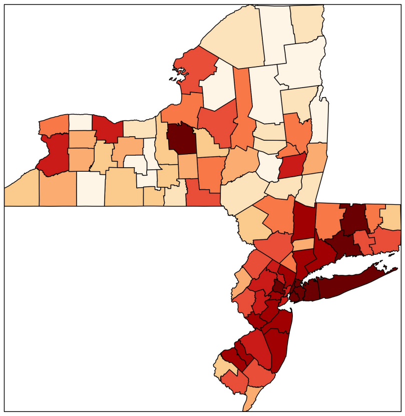

(b) A heatmap of relative infection prevalence estimated via both first and higher connectivity effects [$r_s = 0.871$].

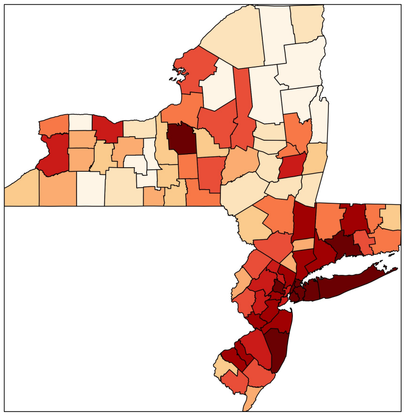

(c) A heatmap of relative infection prevalence estimated via first-order connectivity effects [$r_s = 0.852$].

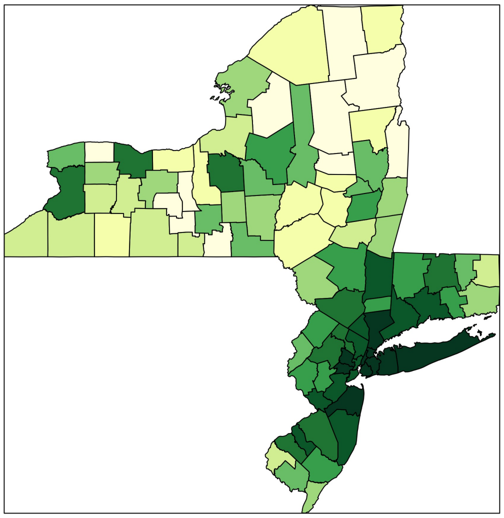

(d) A heatmap of estimated connectivity propagation metric times active caseload.

Name of the county	Rank (by CPM$\times$ active cases)
Queens County	1
Kings County	2
Nassau County	3
Suffolk County	4
Bronx County	5
Westchester County	6
New York County	7
Essex County	8
Monmouth County	9
Union County	10
New Haven County	11
Bergen County	12
Fairfield County	13
Ocean County	14
Middlesex County	15
Richmond County	16
Hudson County	17
Dutchess County	18
Camden County	19
Mercer County	20

(e) List of top twenty counties for likely intervention.

Figure 16. Spatial distributions of estimated relative infection prevalence and observed new COVID-19 cases in the week ending 5 April 2020.

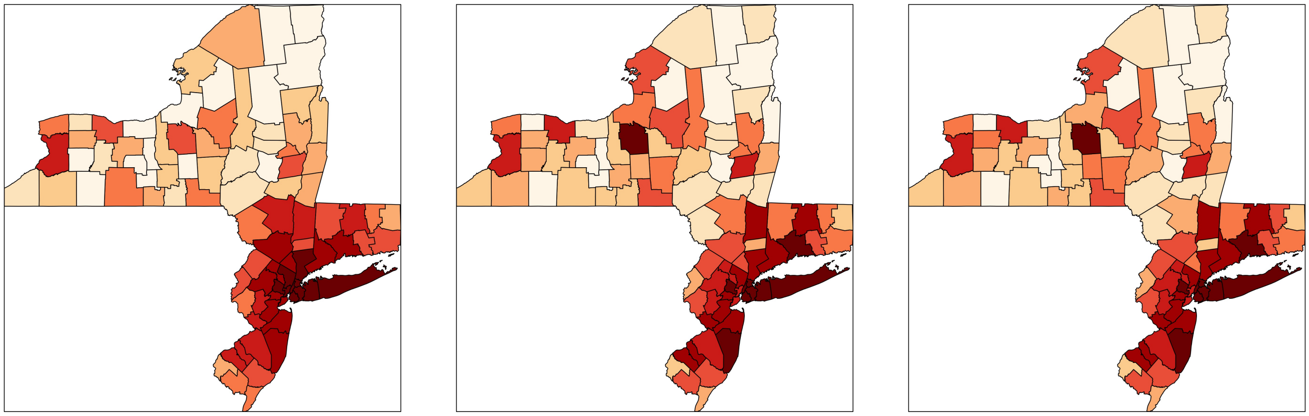

(a) A heatmap of observed cases [Total newly reported cases ≈ 99500].

(b) A heatmap of relative infection prevalence estimated via both first and higher connectivity effects [$r_s = 0.891$].

(c) A heatmap of relative infection prevalence estimated via first-order connectivity effects [$r_s = 0.878$].

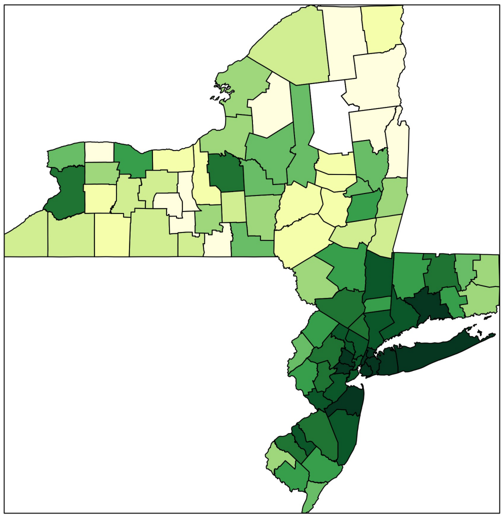

(d) A heatmap of estimated connectivity propagation metric times active caseload.

Name of the county	Rank (by CPM× active cases)
Queens County	1
Kings County	2
Nassau County	3
Suffolk County	4
Essex County	5
Bronx County	6
New Haven County	7
New York County	8
Monmouth County	9
Union County	10
Westchester County	11
Bergen County	12
Middlesex County	13
Ocean County	14
Fairfield County	15
Hudson County	16
Dutchess County	17
Camden County	18
Mercer County	19
Richmond County	20

(e) List of top twenty counties for likely intervention.

Figure 17. Spatial distributions of estimated relative infection prevalence and observed new COVID-19 cases in the week ending 12 April 2020.

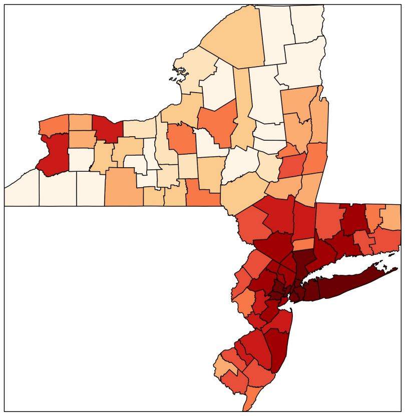

(a) A heatmap of observed cases [Total newly reported cases ≈ 84100].

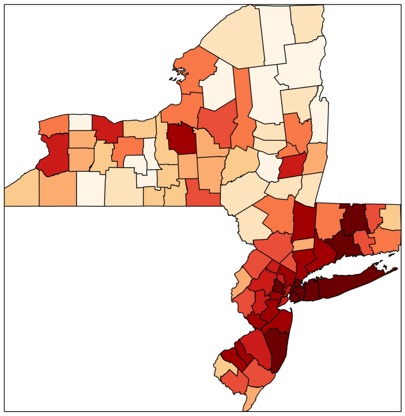

(b) A heatmap of relative infection prevalence estimated via both first and higher connectivity effects [$r_s = 0.870$].

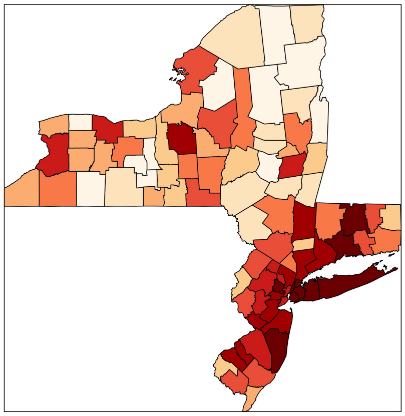

(c) A heatmap of relative infection prevalence estimated via first-order connectivity effects [$r_s = 0.854$].

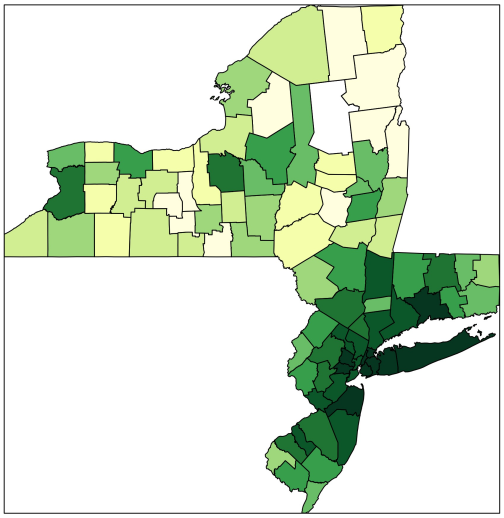

(d) A heatmap of estimated connectivity propagation metric times active caseload.

Name of the county	Rank (by CPM× active cases)
Queens County	1
Kings County	2
Nassau County	3
Suffolk County	4
New Haven County	5
Essex County	6
Bronx County	7
Union County	8
Monmouth County	9
New York County	10
Westchester County	11
Middlesex County	12
Bergen County	13
Fairfield County	14
Ocean County	15
Hudson County	16
Camden County	17
Mercer County	18
Dutchess County	19
Richmond County	20

(e) List of top twenty counties for likely intervention.

Figure 18. Spatial distributions of estimated relative infection prevalence and observed new COVID-19 cases in the week ending 19 April 2020.

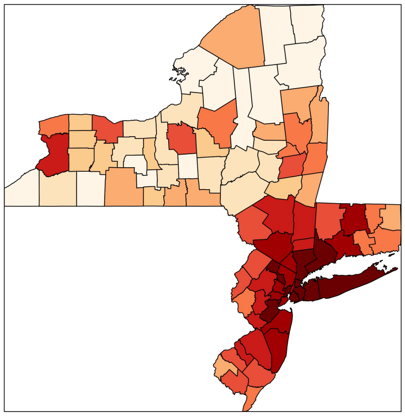

(a) A heatmap of observed cases [Total newly reported cases ≈ 76200].

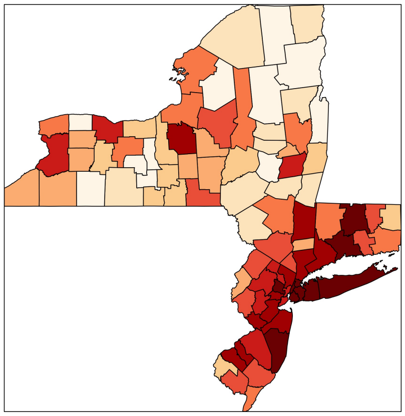

(b) A heatmap of relative infection prevalence estimated via both first and higher connectivity effects [$r_s = 0.850$].

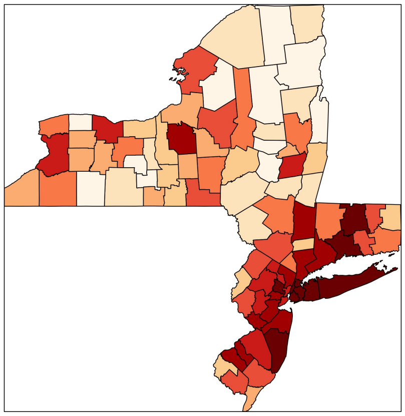

(c) A heatmap of relative infection prevalence estimated via first-order connectivity effects [$r_s = 0.833$].

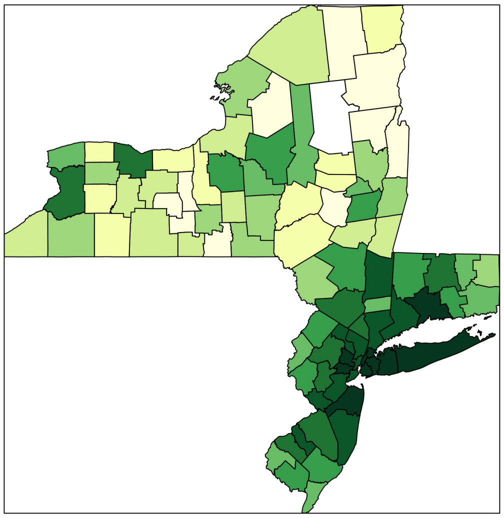

(d) A heatmap of estimated connectivity propagation metric times active caseload.

Name of the county	Rank (by CPM× active cases)
Queens County	1
Kings County	2
New Haven County	3
Suffolk County	4
Nassau County	5
Essex County	6
Bronx County	7
Union County	8
Monmouth County	9
New York County	10
Middlesex County	11
Westchester County	12
Bergen County	13
Fairfield County	14
Ocean County	15
Hudson County	16
Camden County	17
Mercer County	18
Dutchess County	19
Passaic County	20

(e) List of top twenty counties for likely intervention.

Figure 19. Spatial distributions of estimated relative infection prevalence and observed new COVID-19 cases in the week ending 26 April 2020.

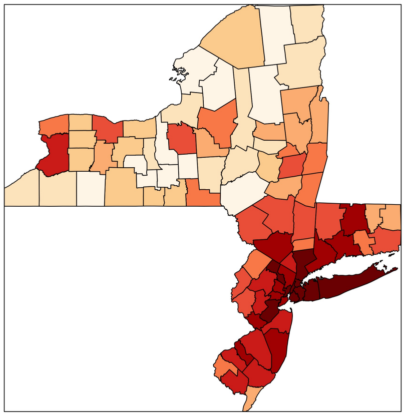

(a) A heatmap of observed cases [Total newly reported cases ≈ 76200].

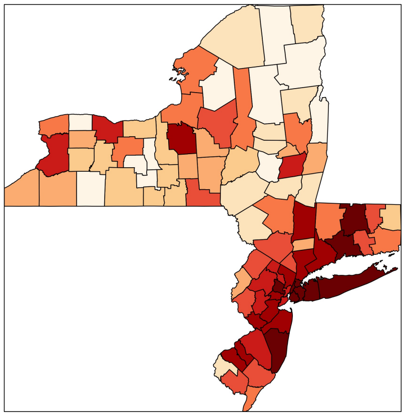

(b) A heatmap of relative infection prevalence estimated via both first and higher connectivity effects [$r_s = 0.857$].

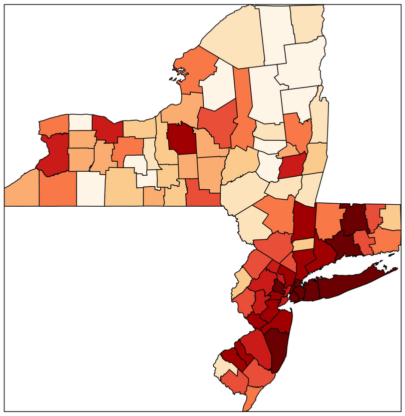

(c) A heatmap of relative infection prevalence estimated via first-order connectivity effects [$r_s = 0.844$].

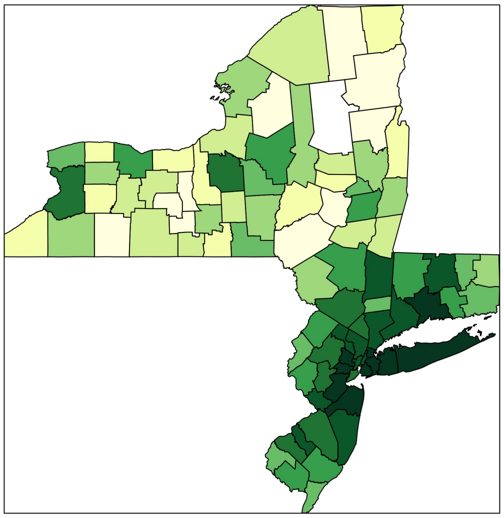

(d) A heatmap of estimated connectivity propagation metric times active caseload.

Name of the county	Rank (by CPM× active cases)
Queens County	1
Kings County	2
New Haven County	3
Suffolk County	4
Nassau County	5
Essex County	6
Bronx County	7
Union County	8
Monmouth County	9
Middlesex County	10
New York County	11
Fairfield County	12
Westchester County	13
Bergen County	14
Ocean County	15
Hudson County	16
Camden County	17
Mercer County	18
Dutchess County	19
Passaic County	20

(e) List of top twenty counties for likely intervention.

Figure 20. Spatial distributions of estimated relative infection prevalence and observed new COVID-19 cases in the week ending 3 May 2020.

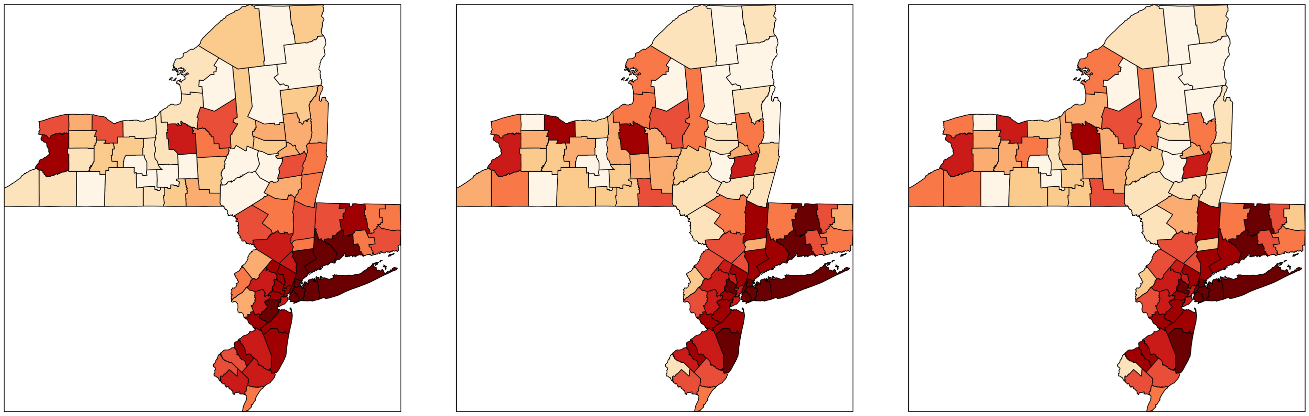

(a) A heatmap of observed cases [Total newly reported cases ≈ 50300].

(b) A heatmap of relative infection prevalence estimated via both first and higher connectivity effects [$r_s = 0.869$].

(c) A heatmap of relative infection prevalence estimated via first-order connectivity effects [$r_s = 0.855$].

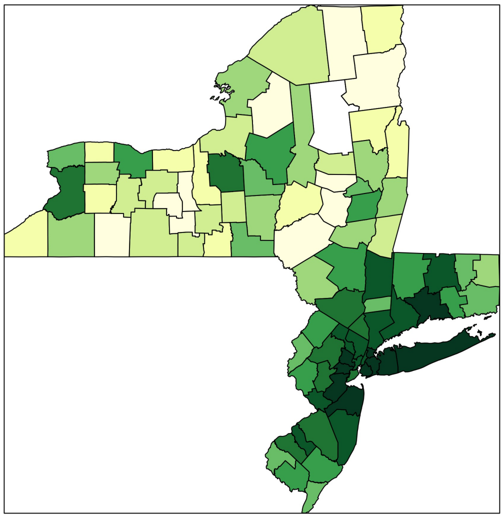

(d) A heatmap of estimated connectivity propagation metric times active caseload.

Name of the county	Rank (by CPM× active cases)
Queens County	1
Kings County	2
New Haven County	3
Suffolk County	4
Essex County	5
Nassau County	6
Bronx County	7
Union County	8
Middlesex County	9
Monmouth County	10
New York County	11
Fairfield County	12
Westchester County	13
Ocean County	14
Bergen County	15
Camden County	16
Hudson County	17
Mercer County	18
Hatford County	19
Passaic County	20

(e) List of top twenty counties for likely intervention.

Figure 21. Spatial distributions of estimated relative infection prevalence and observed new COVID-19 cases in the week ending 10 May 2020.

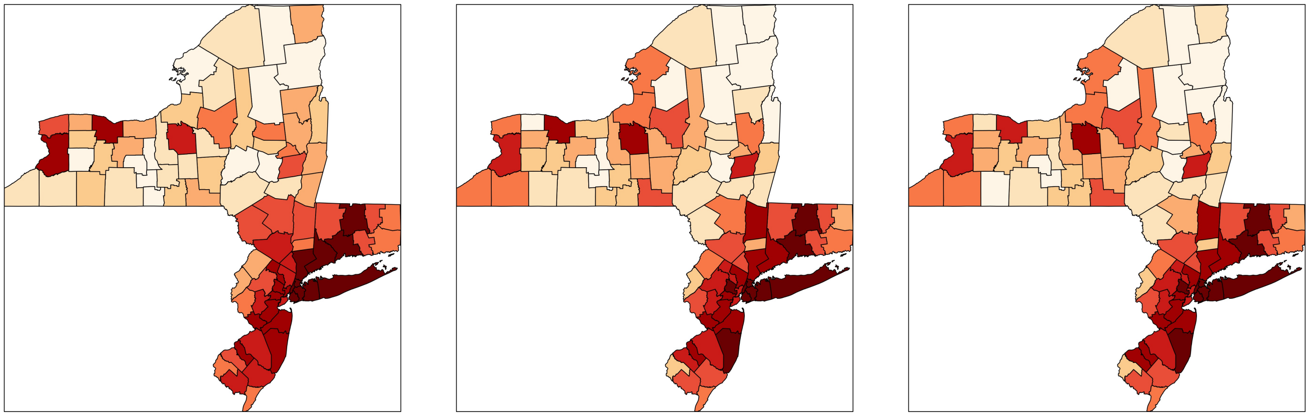

(a) A heatmap of observed cases [Total newly reported cases $\approx$ 35100].

(b) A heatmap of relative infection prevalence estimated via both first and higher connectivity effects [$r_s = 0.878$].

(c) A heatmap of relative infection prevalence estimated via first-order connectivity effects [$r_s = 0.866$].

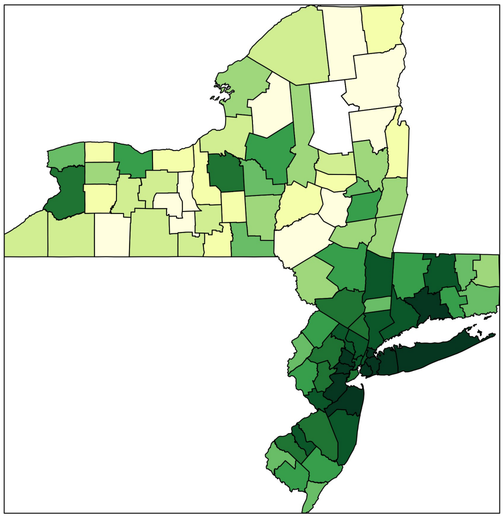

(d) A heatmap of estimated connectivity propagation metric times active caseload.

Name of the county	Rank (by CPM$\times$ active cases)
Queens County	1
Kings County	2
New Haven County	3
Suffolk County	4
Essex County	5
Nassau County	6
Bronx County	7
Union County	8
Middlesex County	9
Monmouth County	10
Fairfield County	11
New York County	12
Westchester County	13
Ocean County	14
Camden County	15
Bergen County	16
Mercer County	17
Hudson County	18
Hartford County	19
Passaic County	20

(e) List of top twenty counties for likely intervention.

Figure 22. Spatial distributions of estimated relative infection prevalence and observed new COVID-19 cases in the week ending 17 May 2020.

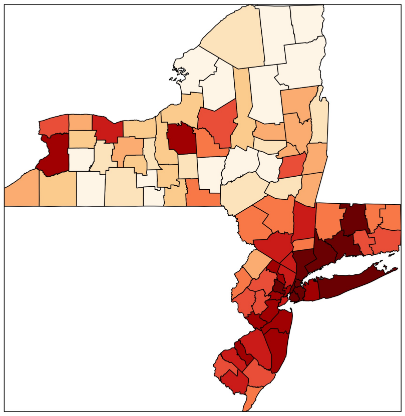

(a) A heatmap of observed cases [Total newly reported cases ≈ 26100].

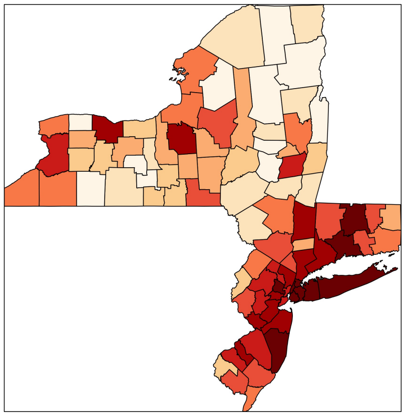

(b) A heatmap of relative infection prevalence estimated via both first and higher connectivity effects [$r_s = 0.899$].

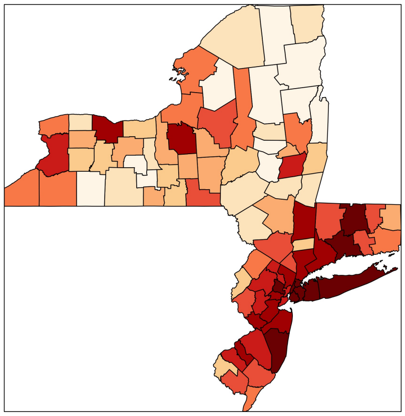

(c) A heatmap of relative infection prevalence estimated via first-order connectivity effects [$r_s = 0.888$].

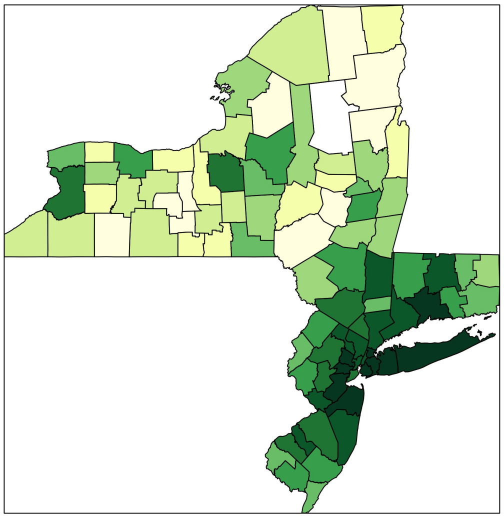

(d) A heatmap of estimated connectivity propagation metric times active caseload.

Name of the county	Rank (by CPM× active cases)
Queens County	1
Kings County	2
New Haven County	3
Suffolk County	4
Essex County	5
Nassau County	6
Bronx County	7
Union County	8
Middlesex County	9
Monmouth County	10
Fairfield County	11
New York County	12
Camden County	13
Ocean County	14
Westchester County	15
Bergen County	16
Mercer County	17
Hudson County	18
Hartford County	19
Passaic County	20

(e) List of top twenty counties for likely intervention.

Figure 23. Spatial distributions of estimated relative infection prevalence and observed new COVID-19 cases in the week ending 24 May 2020.

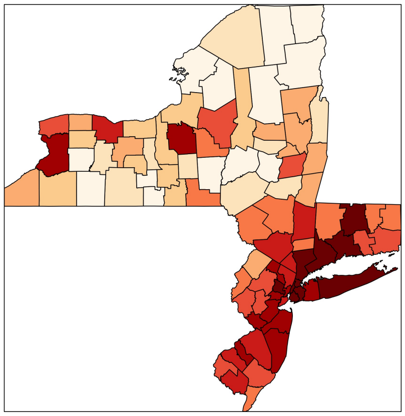

(a) A heatmap of observed cases [Total newly reported cases ≈ 22000].

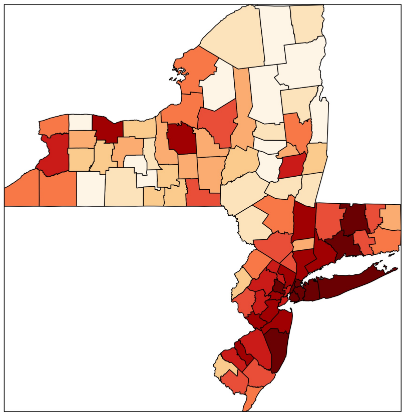

(b) A heatmap of relative infection prevalence estimated via both first and higher connectivity effects [$r_s = 0.896$].

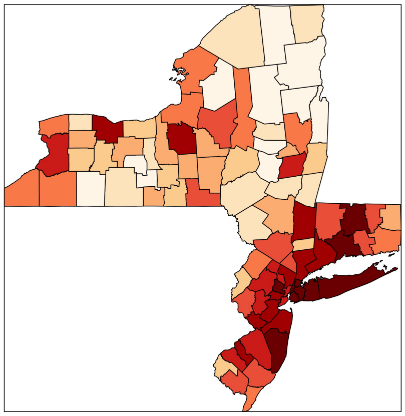

(c) A heatmap of relative infection prevalence estimated via first-order connectivity effects [$r_s = 0.889$].

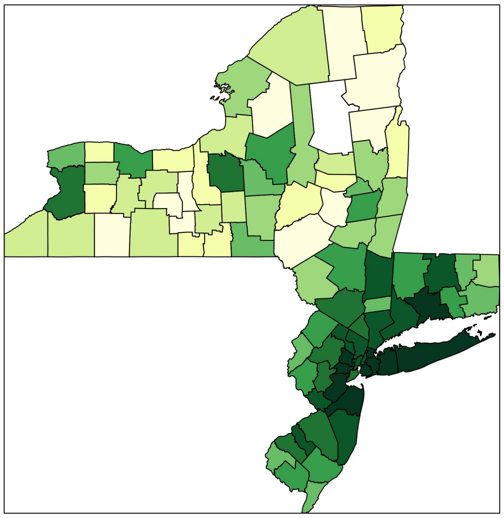

(d) A heatmap of estimated connectivity propagation metric times active caseload.

Name of the county	Rank (by CPM× active cases)
Queens County	1
Kings County	2
New Haven County	3
Essex County	4
Suffolk County	5
Nassau County	6
Bronx County	7
Union County	8
Middlesex County	9
Monmouth County	10
Fairfield County	11
New York County	12
Camden County	13
Ocean County	14
Westchester County	15
Bergen County	16
Mercer County	17
Hudson County	18
Hartford County	19
Dutchess County	20

(e) List of top twenty counties for likely intervention.

Figure 24. Spatial distributions of estimated relative infection prevalence and observed new COVID-19 cases in the week ending 31 May 2020.

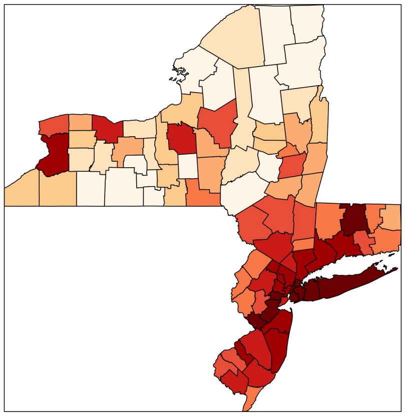

(a) A heatmap of observed cases [Total newly reported cases $\approx$ 12900].

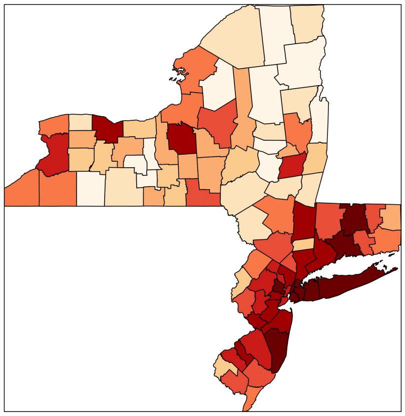

(b) A heatmap of relative infection prevalence estimated via both first and higher connectivity effects [$r_s = 0.895$].

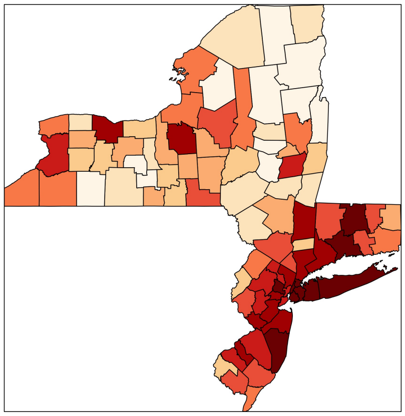

(c) A heatmap of relative infection prevalence estimated via first-order connectivity effects [$r_s = 0.886$].

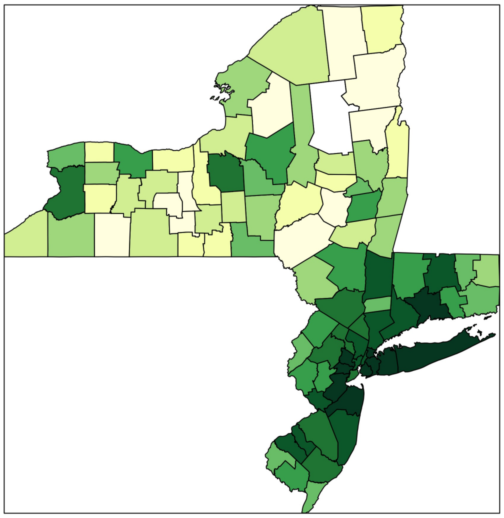

(d) A heatmap of estimated connectivity propagation metric times active caseload.

Name of the county	Rank (by CPM$\times$ active cases)
Queens County	1
Kings County	2
New Haven County	3
Essex County	4
Suffolk County	5
Nassau County	6
Bronx County	7
Union County	8
Middlesex County	9
Monmouth County	10
Fairfield County	11
New York County	12
Camden County	13
Ocean County	14
Westchester County	15
Mercer County	16
Bergen County	17
Hudson County	18
Hartford County	19
Gloucester County	20

(e) List of top twenty counties for likely intervention.

Figure 25. Spatial distributions of estimated relative infection prevalence and observed new COVID-19 cases in the week ending 7 June 2020.

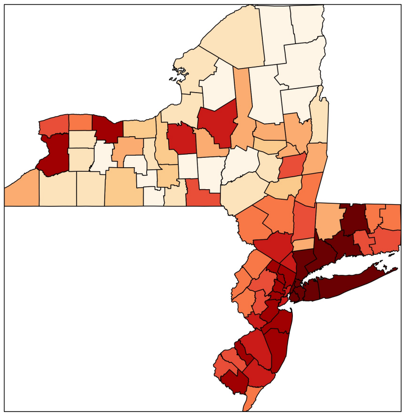

(a) A heatmap of observed cases [Total newly reported cases ≈ 9100].

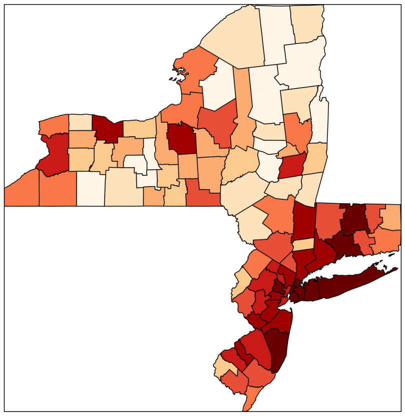

(b) A heatmap of relative infection prevalence estimated via both first and higher connectivity effects [$r_s = 0.909$].

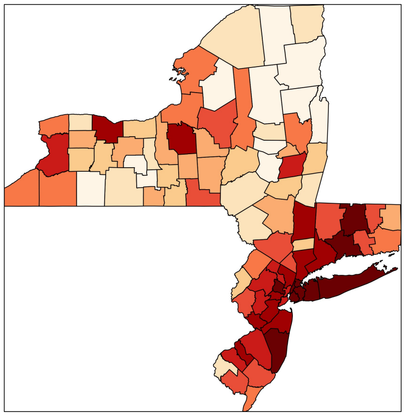

(c) A heatmap of relative infection prevalence estimated via first-order connectivity effects [$r_s = 0.900$].

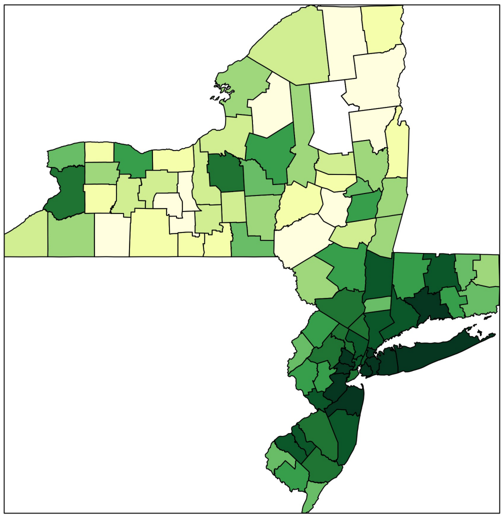

(d) A heatmap of estimated connectivity propagation metric times active caseload.

Name of the county	Rank (by CPM× active cases)
Queens County	1
Kings County	2
New Haven County	3
Essex County	4
Suffolk County	5
Nassau County	6
Bronx County	7
Union County	8
Monmouth County	9
Middlesex County	10
Fairfield County	11
Camden County	12
New York County	13
Ocean County	14
Mercer County	15
Westchester County	16
Bergen County	17
Hudson County	18
Hartford County	19
Gloucester County	20

(e) List of top twenty counties for likely intervention.

Figure 26. Spatial distributions of estimated relative infection prevalence and observed new COVID-19 cases in the week ending 14 June 2020.

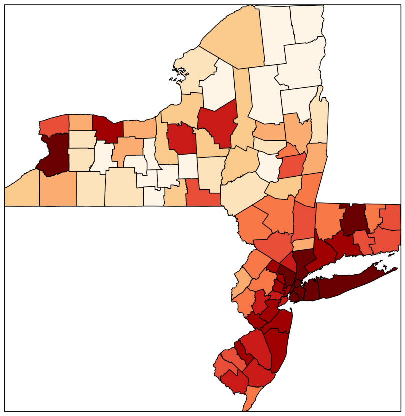

(a) A heatmap of observed cases [Total newly reported cases ≈ 7600].

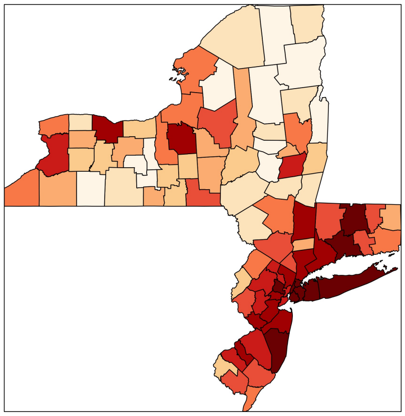

(b) A heatmap of relative infection prevalence estimated via both first and higher connectivity effects [$r_s = 0.828$].

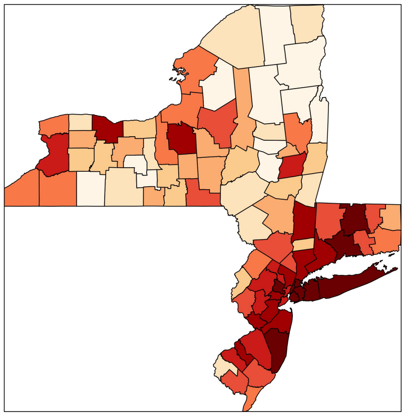

(c) A heatmap of relative infection prevalence estimated via first-order connectivity effects [$r_s = 0.820$].

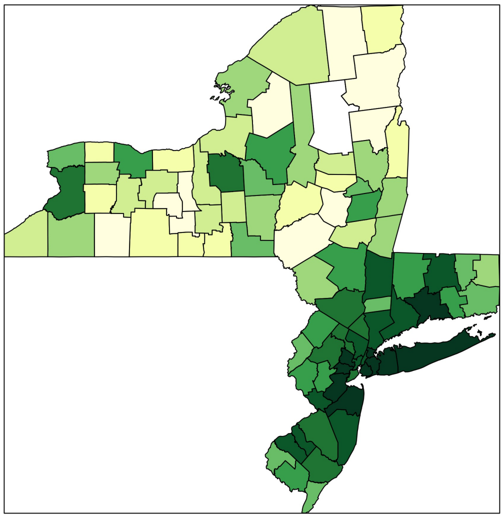

(d) A heatmap of estimated connectivity propagation metric times active caseload.

Name of the county	Rank (by CPM× active cases)
Queens County	1
Kings County	2
New Haven County	3
Essex County	4
Suffolk County	5
Nassau County	6
Bronx County	7
Union County	8
Monmouth County	9
Middlesex County	10
Fairfield County	11
Camden County	12
New York County	13
Ocean County	14
Mercer County	15
Westchester County	16
Bergen County	17
Hudson County	18
Hartford County	19
Gloucester County	20

(e) List of top twenty counties for likely intervention.

Figure 27. Spatial distributions of estimated relative infection prevalence and observed new COVID-19 cases in the week ending 21 June 2020.

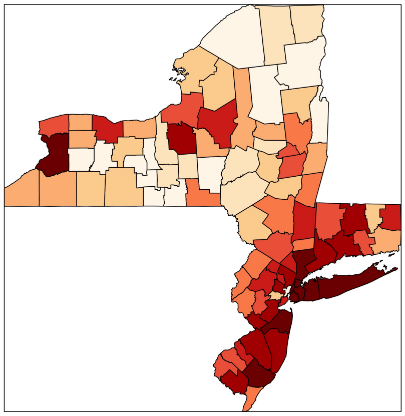

(a) A heatmap of observed cases [Total newly reported cases $\approx$ 7200].

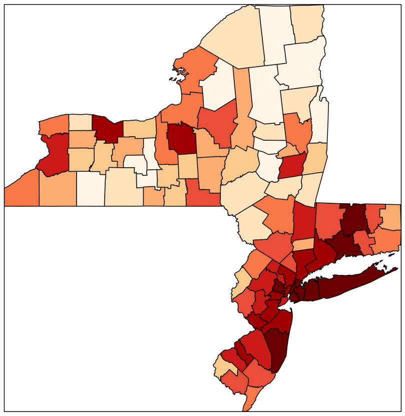

(b) A heatmap of relative infection prevalence estimated via both first and higher connectivity effects [$r_s = 0.853$].

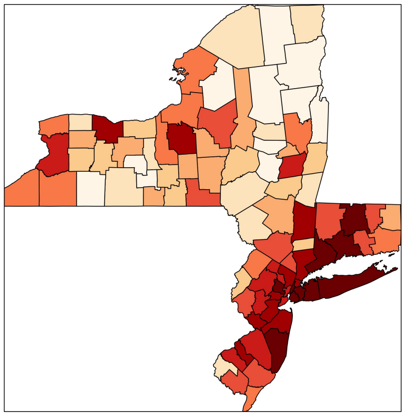

(c) A heatmap of relative infection prevalence estimated via first-order connectivity effects [$r_s = 0.841$].

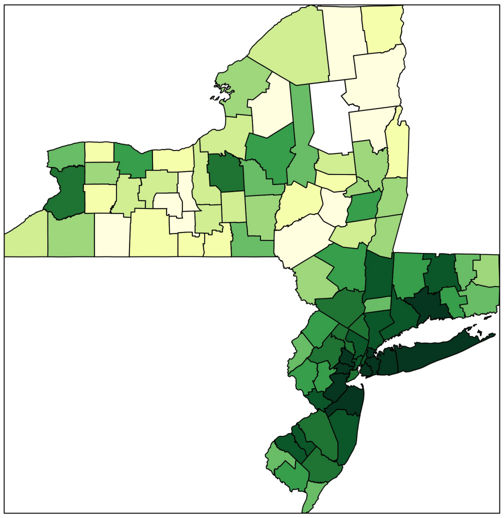

(d) A heatmap of estimated connectivity propagation metric times active caseload.

Name of the county	Rank (by CPM$\times$ active cases)
Queens County	1
Kings County	2
New Haven County	3
Essex County	4
Suffolk County	5
Nassau County	6
Bronx County	7
Union County	8
Monmouth County	9
Middlesex County	10
Fairfield County	11
Camden County	12
New York County	13
Ocean County	14
Mercer County	15
Westchester County	16
Bergen County	17
Hudson County	18
Hartford County	19
Gloucester County	20

(e) List of top twenty counties for likely intervention.

Figure 28. Spatial distributions of estimated relative infection prevalence and observed new COVID-19 cases in the week ending 28 June 2020.
